# Antibacterial insights into alternariol and its derivative alternariol monomethyl ether produced by a marine fungus

**DOI:** 10.1128/aem.00058-24

**Published:** 2024-03-12

**Authors:** Rongmei Li, Zhenjie Su, Chaomin Sun, Shimei Wu

**Affiliations:** 1 College of Life Sciences, Qingdao University, Qingdao, China; 2 CAS Key Laboratory of Experimental Marine Biology, Institute of Oceanology, Chinese Academy of Sciences, Qingdao, China; 3 Center for Deep Sea Research, Institute of Oceanology, Chinese Academy of Sciences, Qingdao, China; 4 Laboratory for Marine Biology and Biotechnology, Qingdao Marine Science and Technology Center, Qingdao, China; 5 Center for Ocean Mega-Science, Chinese Academy of Sciences, Qingdao, China; 6 College of Earth Science, University of Chinese Academy of Sciences, Beijing, China; University of Delaware, Lewes, Delaware, USA

**Keywords:** antibacteria, biosynthesis, alternariol and derivative, marine fungus, multidrug-resistant bacteria

## Abstract

*Alternaria alternata* FB1 is a marine fungus identified as a candidate for plastic degradation in our previous study. This fungus has been recently shown to produce secondary metabolites with significant antimicrobial activity against various pathogens, including methicillin-resistant *Staphylococcus aureus* (MRSA) and the notorious aquaculture pathogen *Vibrio anguillarum*. The antibacterial compounds were purified and identified as alternariol (AOH) and its derivative, alternariol monomethyl ether (AME). We found that AOH and AME primarily inhibited pathogenic bacteria (MRSA or *V. anguillarum*) by disordering cell division and some other key physiological and biochemical processes. We further demonstrated that AOH could effectively inhibit the unwinding activity of MRSA topoisomerases, which are closely related to cell division and are the potential action target of AOH. The antibacterial activities of AOH and AME were verified by using zebrafish as the *in vivo* model. Notably, AOH and AME did not significantly affect the viability of normal human liver cells at concentrations that effectively inhibited MRSA or *V. anguillarum*. Finally, we developed the genetic operation system of *A. alternata* FB1 and blocked the biosynthesis of AME by knocking out *omtI* (encoding an O-methyl transferase), which facilitated *A. alternata* FB1 to only produce AOH. The development of this system in the marine fungus will accelerate the discovery of novel natural products and further bioactivity study.

**IMPORTANCE:**

More and more scientific reports indicate that alternariol (AOH) and its derivative alternariol monomethyl ether (AME) exhibit antibacterial activities. However, limited exploration of their detailed antibacterial mechanisms has been performed. In the present study, the antibacterial mechanisms of AOH and AME produced by the marine fungus *Alternaria alternata* FB1 were disclosed *in vitro* and *in vivo*. Given their low toxicity on the normal human liver cell line under the concentrations exhibiting significant antibacterial activity against different pathogens, AOH and AME are proposed to be good candidates for developing promising antibiotics against methicillin-resistant *Staphylococcus aureus* and *Vibrio anguillarum*. We also succeeded in blocking the biosynthesis of AME, which facilitated us to easily obtain pure AOH. Moreover, based on our previous results, *A. alternata* FB1 was shown to enable polyethylene degradation.

## INTRODUCTION

The marine environment, characterized by its unique ecological conditions, has fostered the evolution of highly adaptive microorganisms. Fungi are a big part of the marine environment (1). To thrive in the competitive and challenging marine milieu, marine fungi produce an array of secondary metabolites endowed with the potential to inhibit various pathogenic microorganisms. Marine fungi produce a range of secondary metabolites with antimicrobial potential, and these secondary metabolites typically represent the chemical defense mechanisms of marine microorganisms (2, 3). Fungal polyketide compounds are important secondary metabolites, and the polyketide synthase in their biosynthetic gene clusters can catalyze the synthesis of structurally complex and diverse compounds, thus possessing significant biological activities (4–9). It is known that fungal secondary metabolites have many biological activities, including antifungal (3, 10), antibacterial (11), anti-inflammatory (12), antiviral (13, 14), and anticancer (15–18).

Alternariol (AOH) and its derivative alternariol monomethyl ether (AME) are diphenylpyranone compounds produced by *Alternaria* fungi and belong to the polyketide class of secondary metabolites (19, 20). AOH was formed through the catalysis of one acetyl-CoA (Ac-CoA) and six malonyl-CoA (Mal-CoA) molecules by the polyketide synthase PksI, followed by the catalysis of 9-hydroxylation to form a methyl ether by O-methyl transferase OmtI, converting AOH into AME (21). To date, only the biosynthesis of AOH and its derivatives has been primarily studied in the terrestrial *Alternaria* fungi (21), while the biosynthesis of these compounds in the marine *Alternaria* species is very lacking.

AOH is usually classified as a toxic metabolite, and it is regarded as an essential contaminant in cereals and fruits (22). The toxicity of AOH has been studied since the 1970s, mainly through *in vitro* models (22). However, insufficient *in vivo* proof has prevented the assessment of AOH health risks for different species, including humans (22). On the contrary, recently, more and more studies have shown that AOH and its derivatives, such as AME, exhibit potential anticancer effects in many pharmacological preclinical test systems (23, 24). AOH and its derivatives exhibit anticancer effects through several pathways, including reactive oxygen species (ROS) generation leading to the induction of oxidative stress and a cytotoxic effect linked to mitochondrial dysfunction (25), inflammatory pathway (26), cell cycle arrest (27), and apoptotic cell death (28). To our knowledge, up to date, no other studies have explored the anticarcinogenic effect of AOH or its derivatives in animal models or clinical trials. Given these promising results of experimental pharmacological studies of AOH and its derivatives, it is reasonable to consider them as potential adjunctive chemotherapeutic agents (29). In addition to anticancer effects, AOH and AME have been shown to possess potential bacteriostasis against several pathogens (19, 30). However, most previous reports only simply checked the antibacterial activity of AOH and AME, and very limited antibacterial mechanisms have been disclosed, hindering their development as effective antibiotics.

Here, focusing on the marine fungus *Alternaria alternata* FB1, which has been identified as a candidate for plastic degradation in our recent study (31), we first showed that it generated AOH and AME that effectively inhibited the growth of methicillin-resistant *Staphylococcus aureus* (MRSA) and *Vibrio anguillarum*. We further detailedly investigated the antibacterial mechanisms of AOH and AME against MRSA and AOH against *V. anguillarum* in combination with electron microscopic observation, transcriptomic analysis, and physiological and biochemical assays. Based on the above results, we speculate that AOH and AME primarily inhibit pathogens by disrupting cell membranes and affecting cell division, ROS production as well as virulence factor generation. We further demonstrated that the topoisomerase of MRSA is one of the potential action targets of AOH. Afterward, we verified the antibacterial activities of AOH and AME *in vivo* by using the zebrafish and validated their biosafety in the human cell line. Finally, we blocked the generation of AME via a genetic method for easy purification of AOH and discussed the biosynthesis of AOH and AME.

## RESULTS

### Purification and structural analysis of antimicrobial substances from the marine fungus *A. alternata* FB1

**Fig 1:**
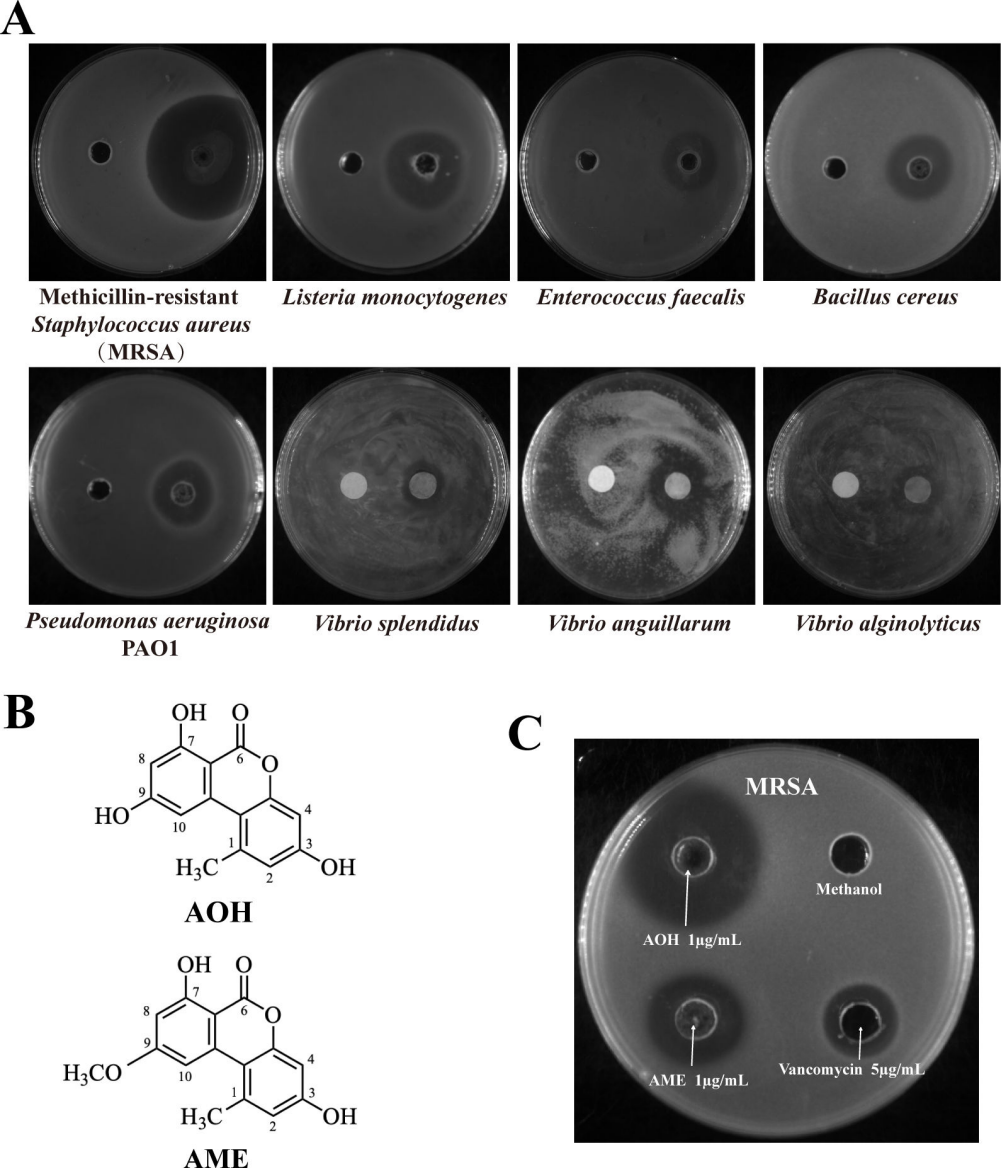
*Alternaria alternata* FB1 produces AOH and AME possessing evident antibacterial effects against methicillin-resistant *Staphylococcus aureus*. (**A**) The fermentation crude extracts of *A. alternata* FB1 resulted clear inhibition zones in the agar plates containing Gram-positive pathogens (including MRSA, *Listeria monocytogenes*, *Enterococcus faecalis*, and *Bacillus cereus*) and Gram-negative pathogens (including *Pseudomonas aeruginosa* PAO1, *Vibrio splendidus*, *Vibrio anguillarum*, and *Vibrio alginolyticus*) as the indicators. (**B**) Structural formulas of AOH and AME. (**C**) Antibacterial activity verification of purified AOH and AME against MRSA. The concentration of each agent used for the assays is as follows: AOH at a concentration of 1 µg/mL, AME at a concentration of 1 µg/mL, and vancomycin at a concentration of 5 µg/mL. Anhydrous methanol, each with 50 µL, was added to the wells of the plate as the negative control.

To evaluate the bacteriostasis potential of metabolites produced by *A. alternata* FB1, its crude extract was obtained (32) and tested for antibacterial activity against four Gram-positive pathogens (including MRSA, *Listeria monocytogenes*, *Enterococcus faecalis*, and *Bacillus cereus*) and four Gram-negative pathogens (including *Pseudomonas aeruginosa* PAO1, *Vibrio splendidus*, *V. anguillarum*, and *Vibrio alginolyticus*). The antibacterial results showed the presence of inhibition zones around the location loaded with crude extract in the plate using the above pathogens as indicators (Fig. 1A), indicating that certain components within the crude extract exhibit inhibitory effects on these pathogenic bacteria. Because MRSA was more significantly inhibited (Fig. 1A), it was chosen as the indicator strain for further investigation. The antibacterial metabolites were sequentially purified by gel filtration chromatography and reverse-phase high-performance liquid chromatography (HPLC). In the HPLC purification step, two antibacterial secondary metabolites were obtained with retention times of 11.944 and 15.584 min (Fig. S1A). Compound 1 and compound 2 were, respectively, used to represent the above two active components and perform structural and functional assays in the present study. Through primary mass spectrometry, the molecular weights of these two substances were determined to be 258.06 Da and 272.08 Da (Fig. S1B), with molecular formulas of C_14_H_10_O_5_ and C_15_H_12_O_5_, respectively.

To elucidate the detailed structure of these two antibacterial compounds, nuclear magnetic resonance (NMR) analysis was further performed. The ^13^C-NMR spectrum of compound 1 indicates the presence of 14 carbon signals in the molecule, including 8 olefinic carbon signals (δ 96.98, 100.95, 101.58, 104.57, 108.96, 117.50, 138.04, and 138.25), 4 oxygenated olefinic carbon signals (δ 152.61, 158.45, 164.06, and 166.13), 1 carbonyl signal (δ 164.68), and 1 methyl signal (δ 25.21). The ^1^H-NMR spectrum of compound 1 shows a methyl proton signal at δ 2.69 (^3^H, s, H-7′), two meta-aromatic proton signals [δ 7.23 (^1^H, d, J = 2.0 Hz, H-6), 6.71 (^1^H, d, J = 2.6 Hz, H-5′), 6.63 (^1^H, d, J = 2.6 Hz, H-3′), and 6.34 (^1^H, d, J = 1.7 Hz, H-4)], and three phenolic hydroxyl signals δ 11.76 (^1^H, s, 4′-OH) and δ 10.66 (^2^H, brs, 3,5-OH) (Fig. S2). Through literature research and structure comparison, it was found that the ^1^H and ^13^C NMR data of this compound are consistent with the reported compound alternariol (33); therefore, compound 1 was identified as alternariol (Fig. 1B).

The ^13^C-NMR spectrum of compound 2 indicates the presence of 15 carbon signals in the molecule, including 8 olefinic carbon signals (δ 98.46, 99.15, 101.64, 103.34, 108.69, 117.66, 137.84, and 138.39), 4 oxygenated olefinic carbon signals (δ 152.65, 158.77, 164.13, and 166.16), 1 carbonyl signal (δ 164.68), 1 methoxy signal (δ 55.82), and 1 methyl signal (δ 25.01). The ^1^H-NMR spectrum shows a methyl proton signal at δ 2.73 (^3^H, s, H-7′), a methoxy proton signal at δ 3.91 (^3^H, s, 5-OCH3), two meta-aromatic proton signals [δ 7.22 (^1^H, d, J = 2.3 Hz, H-6), 6.73 (^1^H, d, J = 2.6 Hz, H-5′), 6.65 (^1^H, d, J = 2.6 Hz, H-3′), and 6.61 (^1^H, d, J = 2.2 Hz, H-4)], and two phenolic hydroxyl signals δ 11.79 (^1^H, s, 4′-OH) and δ 10.52 (^1^H, brs, 3-OH) (Fig. S3). Through literature research and structure comparison, it was found that the ^1^H and ^13^C NMR data of this compound are consistent with the reported compound alternariol-4-methyl ether (19). Therefore, compound 2 was identified as alternariol monomethyl ether (Fig. 1B), the homolog of AOH. The antibacterial activity results showed that AOH and AME could produce an even bigger inhibition zone against MRSA at 1 µg/mL than that of vancomycin at 5 µg/mL (Fig. 1C), indicating their strong potential to develop effective antibiotics.

### Determination of bacteriostasis spectrum and minimum inhibitory concentration of AOH and AME against different pathogenic bacteria

**Fig 2:**
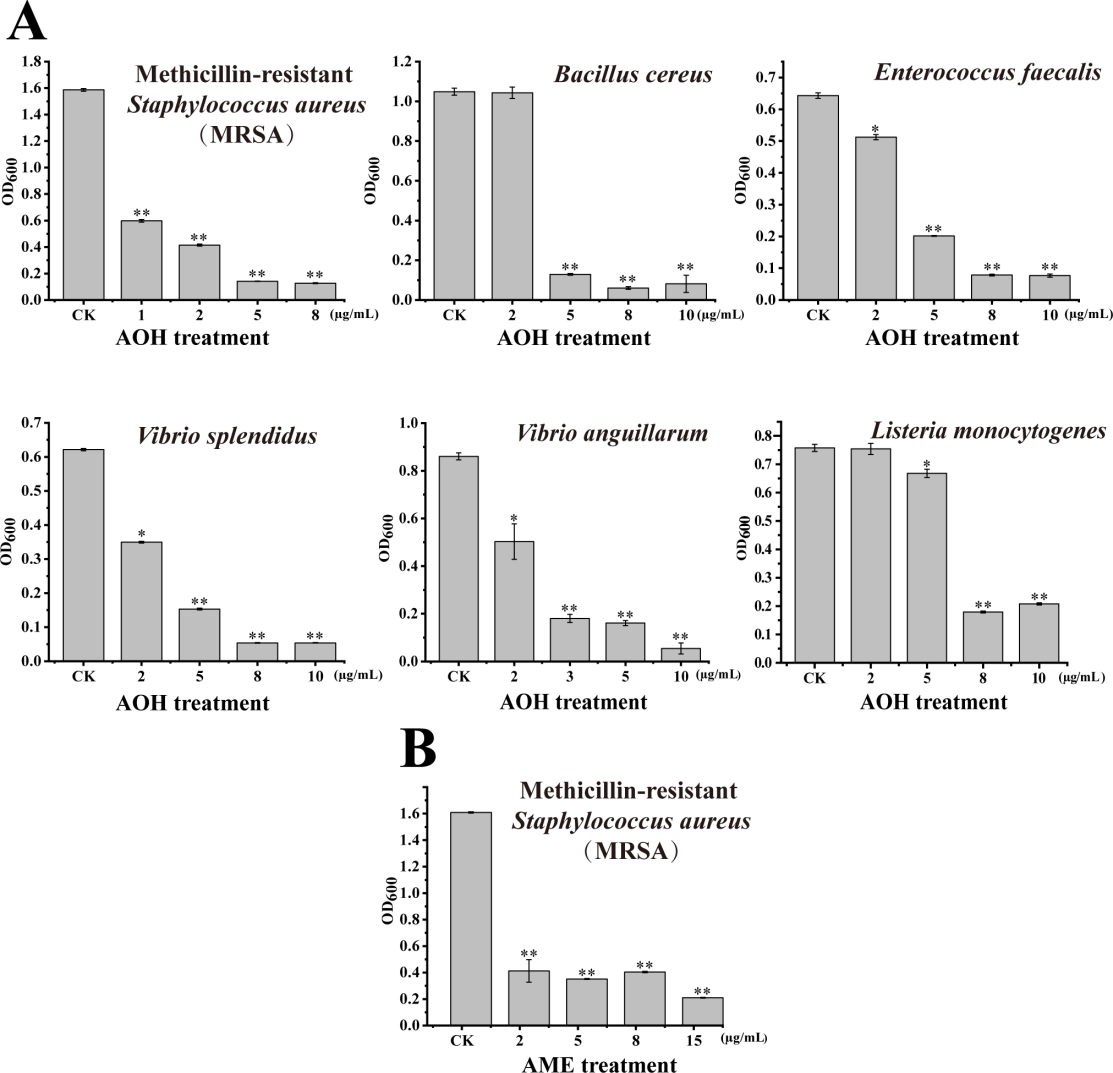
Antibacterial spectrum assays of AOH and AME. (**A**) Antibacterial assays of AOH at different concentrations against Gram-positive and -negative pathogens. The Gram-positive pathogens include MRSA, *Bacillus cereus,* and *Enterococcus faecalis*. The Gram-negative pathogens include *Vibrio splendidus*, *V. anguillarum*, and *L. monocytogenes*. AOH at a final concentration of 1, 2, 5, or 8 µg/mL was used to treat MRSA; AOH at a final concentration of 2, 5, 8, or 10 µg/mL was used to treat *B. cereus*; AOH at a final concentration of 2, 5, 8, or 10 µg/mL was used to treat *E. faecalis*; AOH at a final concentration of 2, 5, 8, or 10 µg/mL was used to treat *L. monocytogenes*; AOH at a final concentration of 2, 5, 8, or 10 µg/mL was used to treat *V. splendidus*; AOH at a final concentration of 2, 3, 5, or 10 µg/mL was used to treat *V. anguillarum*. The growth of MRSA, *Bacillus cereus*, *Enterococcus faecalis,* and *Listeria monocytogenes* was observed after culturing at 37°C, 160 rpm for 24 h, while *V. splendidus* and *V. anguillarum* were cultured at 28°C, 160 rpm for 24 h. An equivalent amount of methanol was used as the negative control. Each group was performed in triplicate and repeated three times. (**B**) Antibacterial assays of AME at different final concentrations against MRSA. AME at a final concentration of 2, 5, 8, or 15 µg/mL was used to treat MRSA. The growth of MRSA was observed after culturing at 37°C, 160 rpm for 24 h. An equivalent amount of methanol was used as the negative control. Each group was performed in triplicate and repeated three times. Error bars represent the standard deviation of three measurements. Differences of *P* ≤ 0.05 were considered statistically significant (*P ≤ 0.05 and **P ≤ 0.01).

To verify the antibacterial spectra of AOH and AME, we conducted antibacterial assays with the same eight pathogenic bacteria used for activity analyses with the crude extract of strain FB1. Among them, AOH showed obvious activity against MRSA, *E. faecalis*, *B. cereus*, *V. splendidus*, *V. anguillarum,* and *L. monocytogenes*. For Gram-positive bacteria, the minimum inhibitory concentrations (MICs) of AOH against MRSA, *B. cereus,* and *E. faecalis* were 5, 5, and 8 µg/mL (Fig. 2A), respectively. Of note, the bacteriostasis rate of AOH against MRSA could reach about 75% at 2 µg/mL (Fig. 2A), suggesting its strong inhibitory activity against MRSA. For Gram-negative bacteria, the MICs of AOH against *V. splendidus*, *V. anguillarum,* and *L. monocytogenes* were 8, 10, and 10 µg/mL (Fig. 2A), respectively. The bacteriostasis rate of AOH against *V. anguillarum* could reach about 82% at 3 µg/mL (Fig. 2A), suggesting its prominent inhibitory activity against *V. anguillarum*. On the other hand, AME only showed an obvious inhibitory effect against MRSA among the above eight testing bacteria (Fig. 2B), and the MIC of AME against MRSA was about 15 µg/mL.

### Ultrastructure and morphological observation of AOH and AME against different pathogenic bacteria

**Fig 3:**
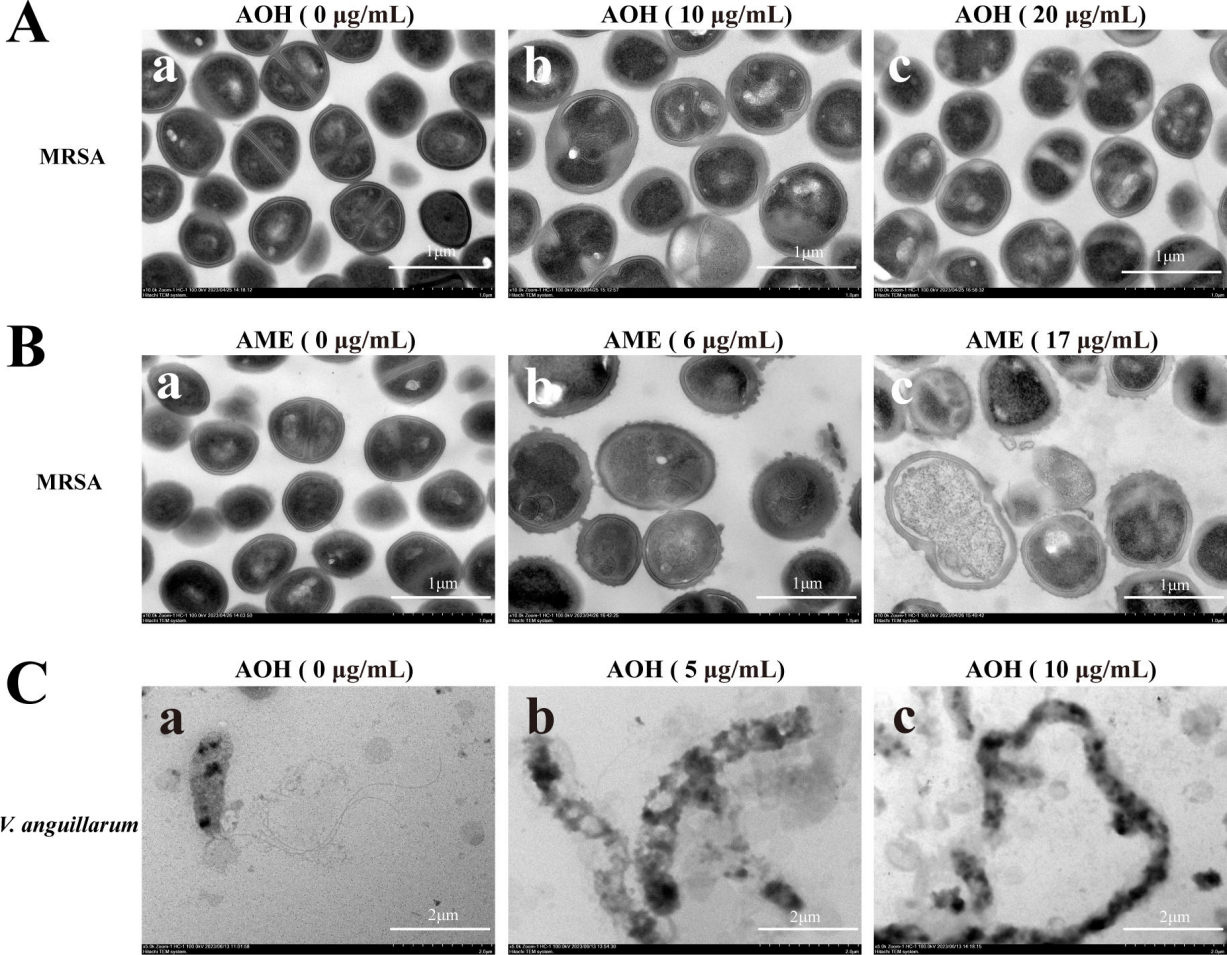
Transmission electron microscope observation of different pathogenic bacteria treated with AOH or AME. (**A**) TEM observation of MRSA treated with different concentrations of AOH. The same amount of methanol was used as a control. (**B**) TEM observation of MRSA treated with different concentrations of AME. The same amount of methanol was used as a control. (**C**) TEM observation of *V. anguillarum* treated with different concentrations of AOH. The same amount of methanol was used as a control. The final concentrations of AOH or AME used for assays are shown above each panel.

To further understand the antibacterial effects of AOH and AME, we observed the morphology changes in MRSA treated with AOH and AME and *V. anguillarum* treated with AOH through scanning electron microscopy (SEM) (Fig. S4) and transmission electron microscopy (TEM) (Fig. 3). The control group was treated with the same amount of methanol, the solvent of AOH and AME. Under SEM observation, the cells of MRSA in the control group showed a very healthy morphology with a round shape and smooth surface (Fig. S4A, panel a and Fig. S4B, panel a); in contrast, MRSA cells treated with a lower concentration of AOH (10 µg/mL) were prone to aggregate (Fig. S4A, panel b), and the cell aggregation became more serious when treated with a higher concentration of AOH (20 µg/mL) (Fig. S4A, panel c); while MRSA cells treated with AME (at 6 and 17 µg/mL) became aggregative and swollen, and part of the cells had been completely broken, with only some fragments gathered together (Fig. S4B, panels b and c). Under TEM observation, the cells of MRSA in the control group showed a very regular inner composition and normal cell division boundary (Fig. 3A, panel a and Fig. 3B, panel a); in contrast, the cell division process of MRSA cells treated with AOH (at 10 and 20 µg/mL) became abnormal, with some bulbous membrane structures formed at the site of division, leading to a very blurry boundary between two divided cells (Fig. 3A, panels b and c); MRSA cells treated with AME (at 6 and 17 µg/mL) showed similar results to those treated with AOH, but the cell surface became more rough and uneven, and cell division disorder became more severe (Fig. 3B, panel b), leading to cell deformation and dissolution, loss of cell integrity, and collapse of the overall cell structure, with leakage of cellular contents (Fig. 3B, panel c). For *V. anguillarum* treated with AOH, SEM observation showed that the cells developed abnormal stretching and aggregation (Fig. S4C, panels b and c); similarly, TEM observation showed that the cell length increased from the original 2 to over 5 µm, and the surface became rough; moreover, most cells lost flagella (Fig. 3C, panels b and c).

### Transcriptome analysis of AOH and AME against pathogens

**Fig 4:**
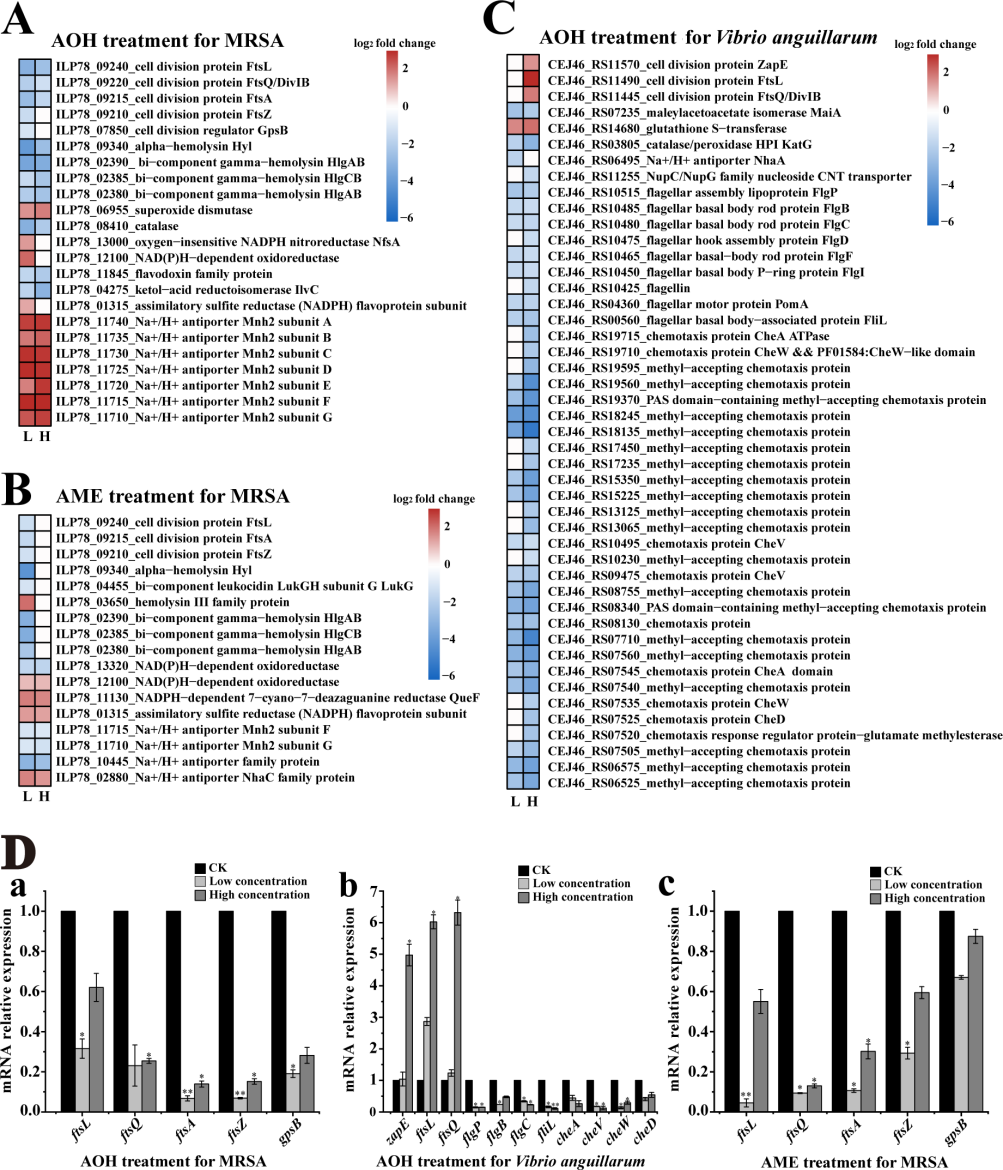
Transcriptomic analyses of pathogenic bacteria treated with AOH and AME. (**A**) Heatmap based on transcriptomics showing significant changes in genes related to cell division, hemolysin synthesis, ROS production, and membrane potential in MRSA treated with AOH. In this panel, “L” indicates the final concentration of AOH used for the treatment of MRSA at 10 µg/mL; “H” indicates the final concentration of AOH used for the treatment of MRSA at 20 µg/mL. (**B**) Heatmap based on transcriptomics showing significant changes in genes related to cell division, hemolysin synthesis, ROS production, and membrane potential in MRSA treated with AME. In this panel, “L” indicates the final concentration of AME used for the treatment of MRSA at 6 µg/mL; “H” indicates the final concentration of AME used for the treatment of MRSA at 17 µg/mL. (**C**) Heatmap based on transcriptomics showing significant changes in genes related to cell division, ROS production, flagella formation, and chemotaxis in *V. anguillarum* treated with AOH. In this panel, “L” indicates the final concentration of AOH used for the treatment of *V. anguillarum* at 5 µg/mL; “H” indicates the final concentration of AOH used for the treatment of *V. anguillarum* at 10 µg/mL. (**D**) Validation of essential genes shown in transcriptome analysis of MRSA treated with AOH and AME and *V. anguillarum* treated with AOH through qRT-PCR. The low- and high concentrations of AOH and AME used for qRT-PCR assays in panels a, b, and c were the same as those in panels A, B, and C, respectively. “CK” indicates the pathogenic bacteria were treated with the same amount of methanol. Differences of *P* ≤ 0.05 were considered statistically significant (*P ≤ 0.05 and **P ≤ 0.01).

To better understand the antibacterial mechanisms of AOH and AME against pathogens, we further investigated the expression changes in genes associated with key life processes of MRSA treated with AOH and AME and *V. anguillarum* treated with AOH. Transcriptome results of MRSA treated by AOH showed that many genes related to cell division and virulence factor hemolysin were significantly downregulated. While membrane Na^+^/H^+^ ions and genes related to ROS were evidently upregulated (Fig. 4A). Transcriptome results of MRSA treated with AME exhibited significant downregulation of many genes related to cell division, the virulence factor hemolysin, and membrane Na^+^/H^+^ ions, while upregulation of genes related to ROS (Fig. 4B). Transcriptome results of AOH-treated *V. anguillarum* showed significant upregulation of many genes related to cell division and ROS, while evident downregulation of many genes related to flagella and chemotaxis (Fig. 4C). Of note, many significantly regulated genes of MRSA and *V. anguillarum* after AOH or AME treatment were associated with cell division, which is consistent with the electron microscopic results (Fig. 3).

To verify the accuracy of transcriptome results, we further used real-time quantitative PCR (qRT-PCR) to detect the expression changes in genes at the transcriptional level in MRSA and *V. anguillarum,* which were treated with same concentrations of AOH or AME. Five selected representative genes related to cell division in MRSA (including *ftsL, ftsQ, ftsA, ftsZ,* and *gpsB*) were downregulated after treatment with AOH (Fig. 4D, panel a) and AME (Fig. 4D, panel c), respectively. For *V. anguillarum* treated with AOH, the genes (including *zapE, ftsL,* and *ftsQ*) involved in cell division (Fig. 4D, panel b) were upregulated by about five to six times; genes related to flagellar formation (including *flgP, flgB, flgC,* and *fliL*) and genes related to chemotaxis (including *cheA, cheV, cheW,* and *cheD*) were significantly downregulated. Together with the transcriptome results, we conclude that the transcriptome and qRT-PCR data are consistent, which also validates the accuracy of the transcriptome results.

### Effects of AOH and AME on the key physiological and biochemical processes of pathogenic bacteria

**Fig 5:**
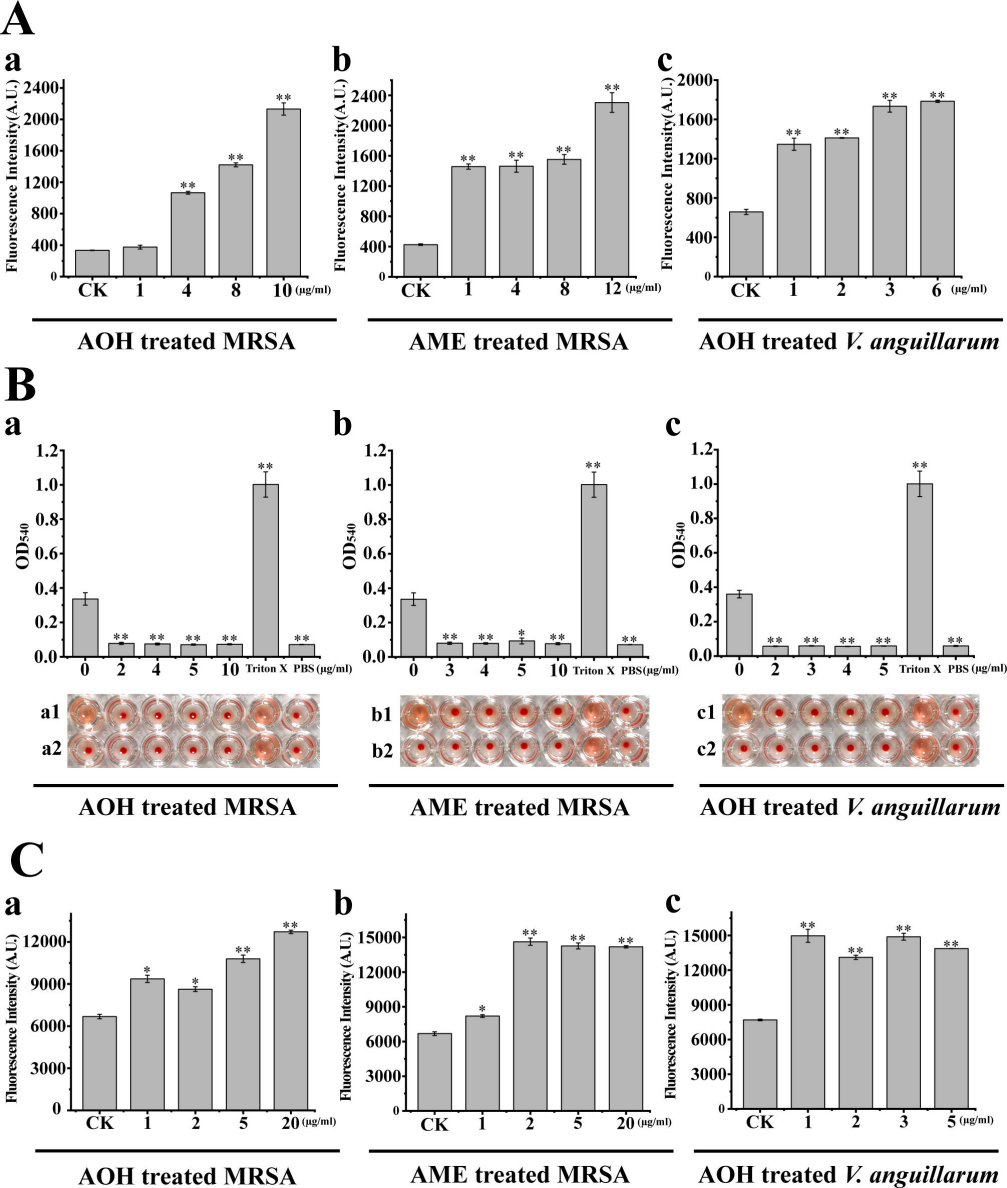
Effects of AOH and AME on the membrane potential, hemolysin generation, and ROS production in different pathogenic bacteria. (**A**) Changes in propidium iodide (indicating the membrane potential) in MRSA treated with different concentrations of AOH or AME and in *V. anguillarum* treated with different concentrations of AOH. The same amount of methanol was used as a control (indicated with “CK”). (**B**) Changes in hemolysin generation in MRSA treated with different concentrations of AOH or AME and in *V. anguillarum* treated with different concentrations of AOH. In panel a, line a1 shows the hemolytic effects of MRSA treated with different concentrations of AOH or the same amount of methanol. In panel b, line b1 shows the hemolytic effects of MRSA treated with different concentrations of AME or the same amount of methanol. In panel c, line c1 shows the hemolytic effects of *V. anguillarum* treated with different concentrations of AOH or the same amount of methanol. Meanwhile, the blood cells treated with the same amount of Triton X and phosphate-buffered saline buffer acted as positive and negative control groups, respectively. The data used for generating histograms in panels a, b, and c were, respectively, derived from the quantitative results shown in line a1, line b1, and line c1, which indicate the hemolytic abilities of corresponding agents added in blood cells. Lines a2, b2, and c2, respectively, shows the hemolytic effects of the same concentrations of AOH, AME, and AOH used in lines a1, b1, and c1. (**C**) Changes in ROS production in MRSA treated with different concentrations of AOH or AME and in *V. anguillarum* treated with different concentrations of AOH. The same amount of methanol was used as a control (indicated with “CK”). Differences of *P* ≤ 0.05 were considered statistically significant (*P ≤ 0.05 and **P ≤ 0.01).

To further verify the antibacterial insights into AOH and AME against pathogenic bacteria disclosed by morphologic and transcriptomic analyses, we detected the changes in various key physiological and biochemical characteristics of MRSA and *V. anguillarum* treated with AOH or AME. We first detected the membrane integrity of MRSA and *V. anguillarum* treated with AOH, as well as MRSA treated with AME through propidium iodide (PI) staining. The results showed that MRSA cells treated with AOH (Fig. 5A, panel a) or AME (Fig. 5A, panel b) exhibited significantly increased PI levels. Especially, MRSA showed a sharp increase in PI intensity at a final concentration of 1 µg/mL of AME, while the AOH-treated cells only showed a gradual increase (Fig. 5A, panels a and b). These results indicated that the cell membranes of MRSA were damaged by the treatment of AOH and AME, and AME could result in an even more harmful effect on the cell membrane at a lower concentration (e.g., 1 µg/mL) than that of AOH. Similarly, the treatment of AOH also markedly enhanced the PI intensity in *V. anguillarum* cells (approximately twice that of the control group; Fig. 5A, panel c). The above results suggested that both AOH and AME might inhibit pathogenic bacteria by disrupting the cell membrane.

It is known that *S. aureus* primarily produces hemolysins, including α-hemolysin (Hla) and β-hemolysin (Hlb) (34, 35), which are key virulence factors determining the severity of *S. aureus* infections. Moreover, hemolysin is also the main cause of septicemia in *V. anguillarum* (36). Therefore, we next checked the production of hemolysins in MRSA and *V. anguillarum* that were treated with AOH or AME. The results of sheep blood lysis showed that the production of hemolysin in MRSA cells treated with AOH (Fig. 5B, panel a) or AME (Fig. 5B, panel b) and *V. anguillarum* cells treated with AOH (Fig. 5B, panel c) was significantly reduced. Especially, the production of hemolysin in MRSA and *V. anguillarum* cells was almost completely inhibited by low concentrations of AOH or AME (e.g., 2–3 µg/mL).

On the other hand, ROS levels play an important role in controlling cellular physiology (37, 38), and detection of intracellular ROS level is essential for judging the effectiveness and understanding potential mechanisms of antibiotics (39). Moreover, the transcriptome results indicated that the expression of genes encoding key proteins related to ROS production was evidently regulated (Fig. 4). We next sought to detect the production levels of ROS in MRSA and *V. anguillarum* cells treated with different concentrations of AOH or AME. The results showed that the generation of ROS was significantly increased in MRSA cells treated with AOH (Fig. 5C, panel a) or AME (Fig. 5C, panel b), as well as in *V. anguillarum* cells treated with AOH (Fig. 5C, panel c).

### Determination of the potential action target of AOH against MRSA

Given that AOH and AME both showed significant inhibition effect on MRSA, we thus chose AOH as the antibacterial metabolite and MRSA as the pathogenic bacterium to study the potential action target of AOH. Through electron microscopic (Fig. 3) and transcriptomic (Fig. 4) analyses, we inferred that AOH profoundly affects the cell division of MRSA and *V. anguillarum*. With that, we speculate that AOH might impact the key enzymes linked to cell division. Indeed, some research indicated that AOH might inhibit the activity of topoisomerase (40–42), which is crucial for the bacterial cell division process. We next sought to ask whether AOH could hinder the unwinding activity of MRSA topoisomerases. On combining the transcriptomic results and the genome of MRSA, we found genes (including *ILP78_00775*, *ILP78_00780*, *ILP78_08890,* and *ILP78_03260*) potentially encoding topoisomerases, which were, respectively, named Top1, Top2, Top3, and Top4 in the present study. Top1 and Top2 could potentially be two separate subunits of a single complex, where Top2 alone does not exhibit activity (43). We therefore combined Top1 and Top2 together and named as Topc here.

**Fig 6:**
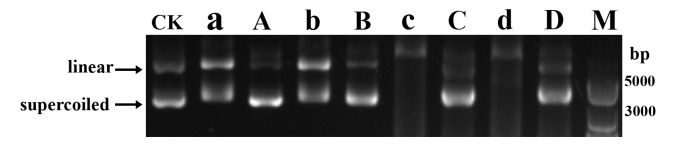
AOH effectively inhibited the unwinding activities of topoisomerases of MRSA. The supercoiled form was effectively unwound to the liner one when the plasmid pCT74 was treated with topoisomerases Top1 (lane a), Topc (lane b), Top3 (lane c), or Top4 (lane d) at 37°C for 30 min. However, the unwinding activity of topoisomerases Top1 (lane A), Topc (lane B), Top3 (lane C), or Top4 (lane D) was effectively inhibited when the above topoisomerases were pre-incubated with AOH (at a final concentration of 4 µg/mL) at 37°C for 5 min before incubating with pCT74 for another 30 min. “CK” represents only protein storage buffer was used for unwinding assays. “M” indicates the DNA marker. Each reaction volume is 20 µL. For each reaction, 1.2 µg of pCT74 and 8 µM (final concentration) of different topoisomerases were added. 1.5% agarose gel electrophoresis was performed to check the linear or supercoiled forms of pCT74.

Next, we conducted *in vitro* expression of the above topoisomerases in *Escherichia coli* and analyzed their unwinding activities toward the plasmid pCT74 in the absence or presence of AOH. As a negative control, the plasmid pCT74 was treated with the storage buffer of topoisomerase. The results showed that, compared to the negative control, the intensity of supercoiled bands of pCT74 became much higher when treated with Top1 and Topc, and the supercoiled bands of pCT74 even disappeared after treatment with Top3 and Top4 (Fig. 6). The above results strongly indicated that Top1, Topc, Top3, and Top4 had evident unwinding activities, which changed the supercoiled form of pCT74 into a linear form. However, when we pre-incubated AOH at a final concentration of 4 µg/mL with Top1, Topc, Top3, and Top4 at 37°C for 5 min before treating pCT74, we found the amount of linear form of pCT74 was much less than that of the supercoiled state (Fig. 6), indicating AOH effectively blocked the unwinding activities of the above four topoisomerases. As topoisomerases are closely related to bacterial DNA replication and cell division (44), we speculate that topoisomerase might be one of the key action targets of AOH against MRSA.

### Verification of antibacterial activities of AOH and AME *in vivo* and biosafety assays of AOH and AME in human cell line

**Fig 7:**
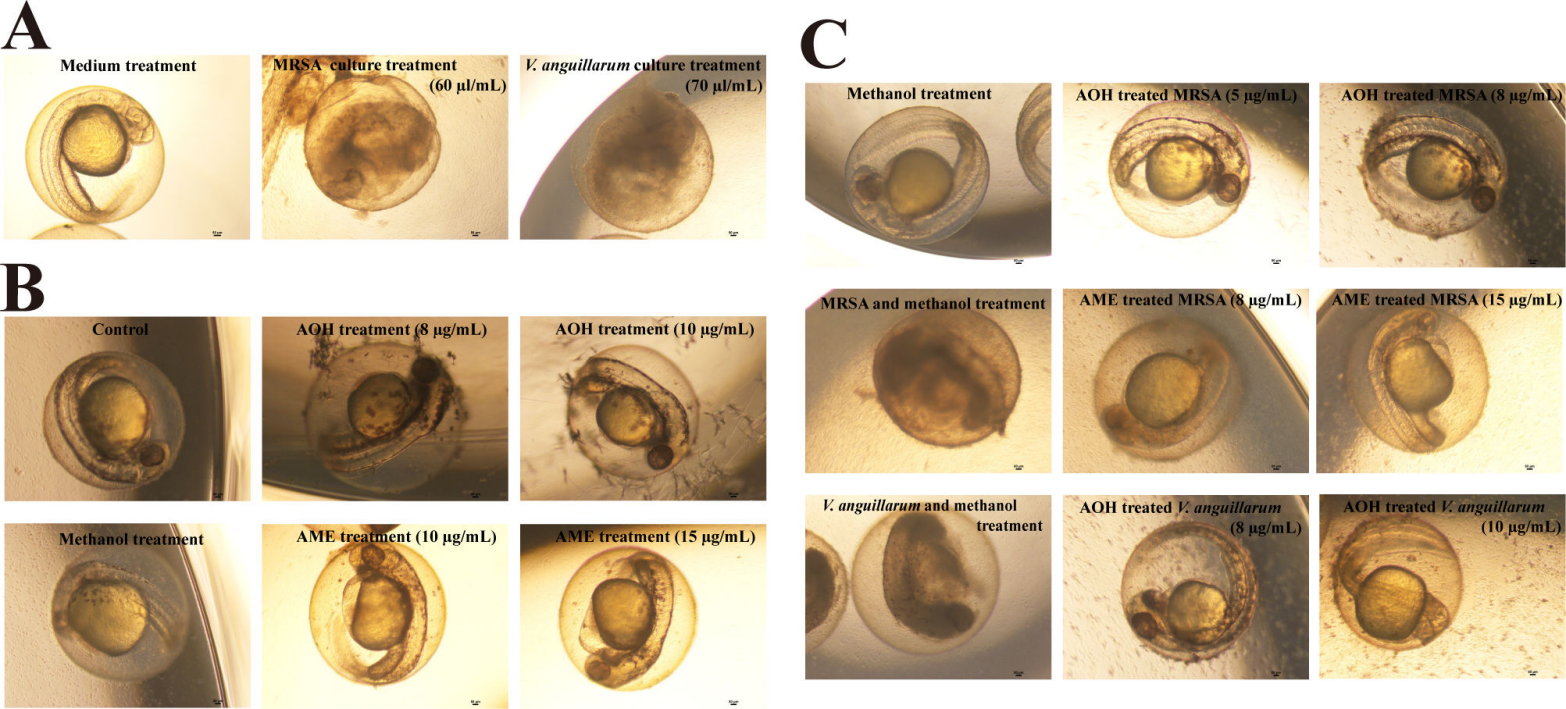
Verification of antibacterial activities of AOH and AME against pathogenic bacteria by using zebrafish as the *in vivo* model. (**A**) Representative pictures showing the harmful effects of MRSA and *V. anguillarum* on the development of zebrafish embryos. For MRSA, 60 µL of overnight cultured cells was added to the well containing 1 mL of embryo water and healthy zebrafish embryos and incubated at 28°C for 24 h; 60 µL of LB medium was used as a negative control. For *V. anguillarum*, 70 µL of overnight cultured cells was added to the well containing 1 mL of embryo water and healthy zebrafish embryos and incubated at 28°C for 24 h; 70 µL of 2216E medium was used as a negative control. (**B**) Representative pictures showing the effects of different concentrations of AOH (8 and 10 µg/mL) and AME (10 and 15 µg/mL) on the development of zebrafish embryos. The zebrafish embryos were imaged after a 24-h incubation with AOH or AME at 28°C. The same amount of methanol was used as the negative control. (**C**) Representative pictures showing the protection of AOH and AME against the harmful effects of MRSA and *V. anguillarum* on the development of zebrafish embryos. The zebrafish embryos were imaged after a 24-h incubation with AOH or AME at 28°C. The same amount of methanol was used as the negative control.

To evaluate the pharmacability of AOH and AME, we checked their antibacterial activities by using zebrafish as the *in vivo* model. To this end, we selected zebrafish embryos within 24-h post-fertilization for further *in vivo* assays. First, we tested the toxic effects of MRSA and *V. anguillarum* on the development of zebrafish embryos. The results showed that the supplement of overnight cultured bacterial cells of MRSA (60 µL/mL) or *V. anguillarum* (70 µL/mL) led to zebrafish embryos being incomplete and deformed (Fig. 7A), indicating the above pathogenic bacteria could significantly affect the development of zebrafish embryos. Given that AOH and AME could evidently inhibit the growth of MRSA or *V. anguillarum*, we next explored whether AOH or AME could prevent zebrafish embryos from infection by the above two pathogenic bacteria. We first checked whether AOH or AME alone could result in harmful effects on zebrafish embryos at the concentrations that showed significant inhibitory effects on MRSA or *V. anguillarum* as described in the present study. The results showed that there was no negative impact on the development of zebrafish embryos under the concentrations of AOH (8 and 10 µg/mL) and AME (10 and 15 µg/mL) inhibiting pathogenic bacteria (Fig. 7B). Notably, MRSA (60 µL/mL) or *V. anguillarum* (70 µL/mL) cells did not result in any harmful effects on the development of zebrafish embryos in the presence of AOH and AME at the concentrations used for *in vitro* antibacterial assays (Fig. 7C). In contrast, the development of zebrafish embryos in the negative control groups became much abnormal (Fig. 7C). The above results clearly demonstrated that AOH and AME could effectively inhibit the infection of pathogenic bacteria toward zebrafish embryos, which strongly indicates the antibacterial functions of AOH and AME work well *in vivo*.

**Fig 8:**
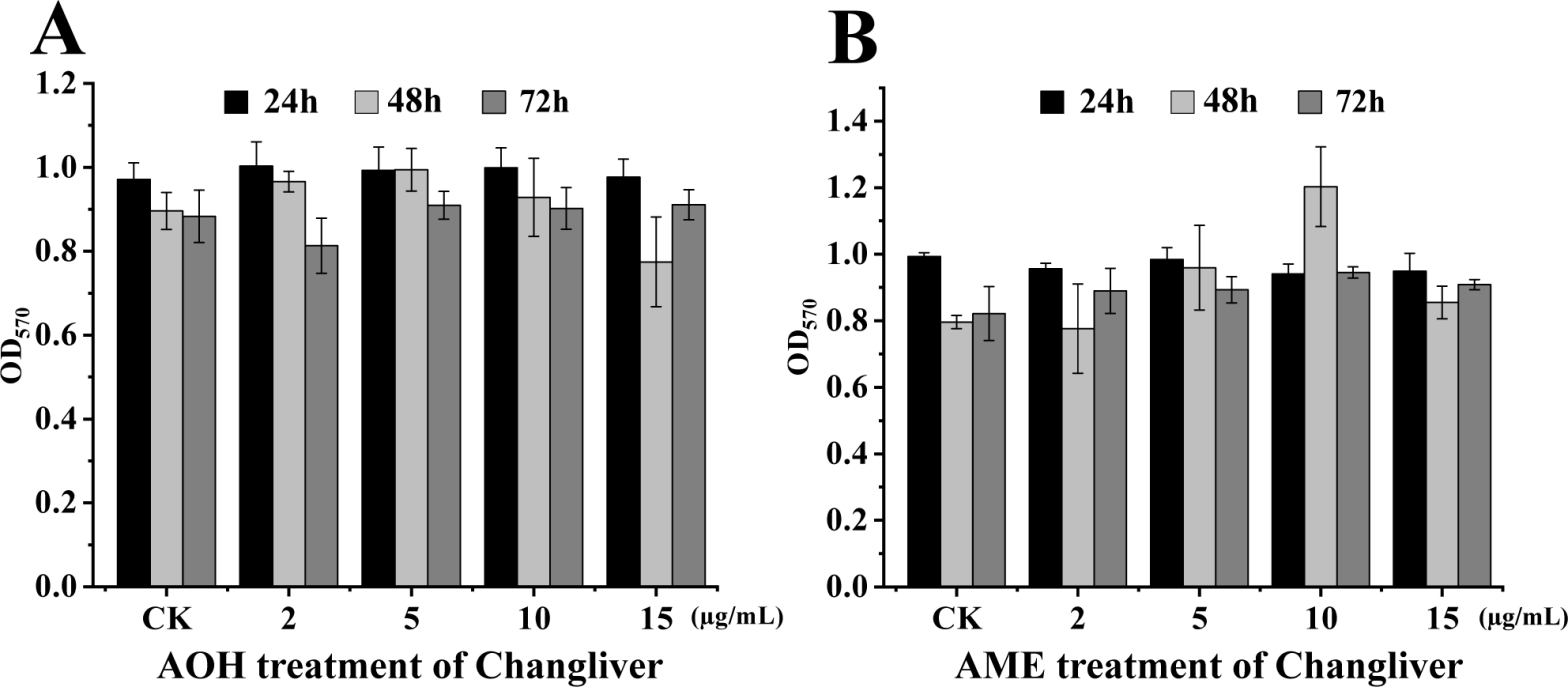
Effects of AOH and AME on human normal liver cell line (Chang liver). (**A**) MTT assays of Chang liver cells treated with different concentrations of AOH. OD_570_ values indicate the viability of Chang liver cells. The same amount of methanol was used for the negative control. (**B**) MTT assays of Chang liver cells treated with different concentrations of AME. OD_570_ values indicate the viability of Chang liver cells. The same amount of methanol was used for the negative control.

The difference in the toxic effect of a drug candidate on pathogens and human cells is critical to evaluate its druggability, and the normal human cell line Chang liver is often used to evaluate the toxicity of a drug candidate (45). In this study, Chang liver cells were treated with different concentrations of AOH (Fig. 8A) or AME (Fig. 8B), and MTT assay was performed. After culture for 24, 48, and 72 h, it was found that the survival rate of Chang liver cells treated with AOH (Fig. 8A) or AME (Fig. 8B) did not change significantly compared with the control group. Importantly, even at a final concentration at 15 µg/mL of AOH or AME (which significantly inhibited the tested pathogens), the viability of Chang liver cells was not evidently changed compared to that of control, suggesting the safety of AOH or AME to be developed as potential antibiotics in the future.

### Verification of key genes responsible for biosynthesis of AOH and AME

**Fig 9:**
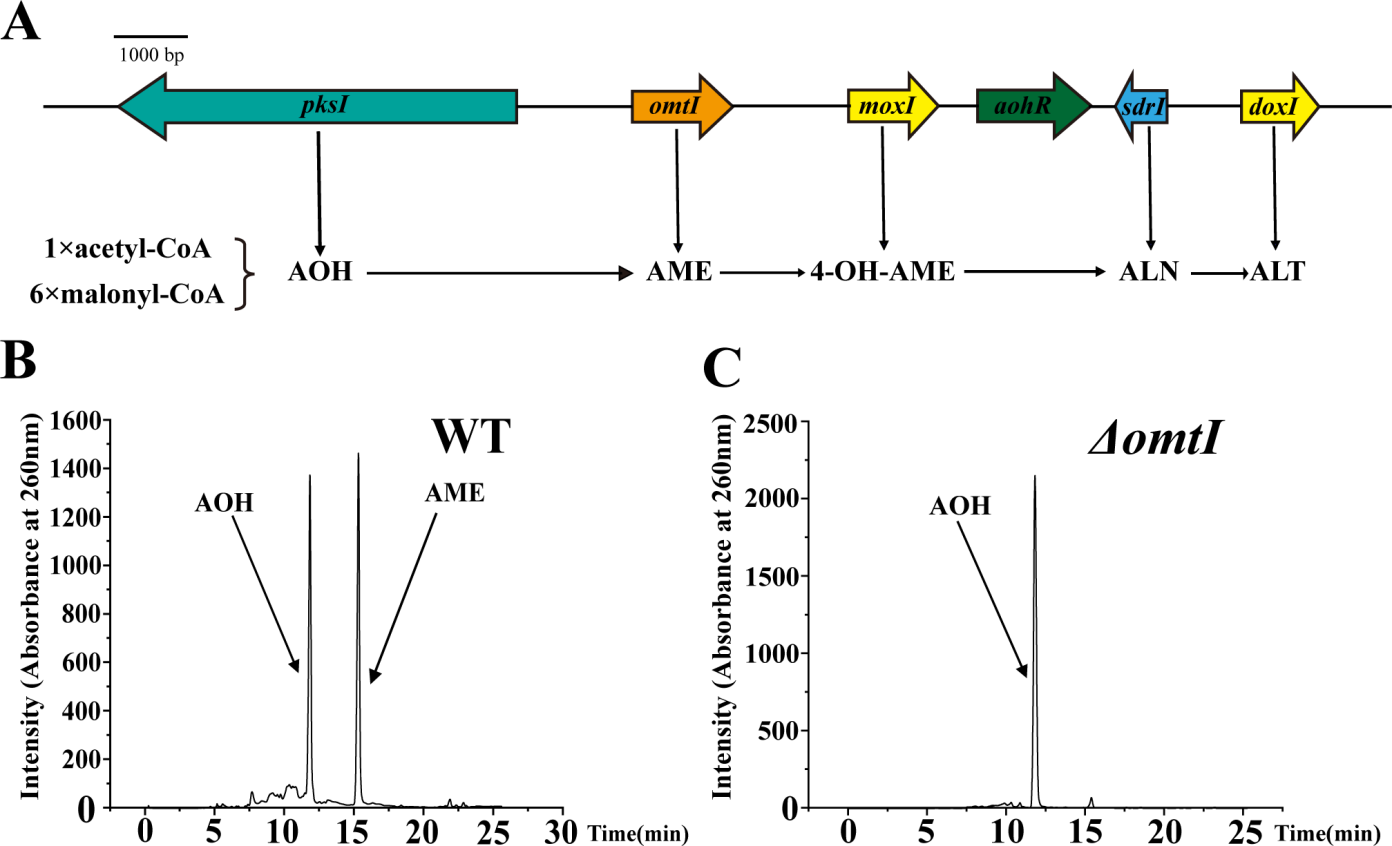
Deletion of *omtI* blocks the biosynthesis of AME. (**A**) The gene cluster directing the biosynthesis of AOH and AME identified in *A. alternata* FB1. The function of each gene directing the biosynthesis of AOH and its derivatives was also accordingly indicated. PksI catalyzes one acetyl-CoA (Ac-CoA) and six malonyl-CoA (Mal-CoA) units into heptaketide AOH. OmtI catalyzes the formation of a methyl ether through 9-hydroxylation, transforming AOH into AME. MoxI catalyzes hydroxylation at the 4-position of AME, forming 4-OH-AME. SdrI initiates the lactone ring formation, resulting in ALN. Additionally, DoxI might catalyze the generation of ALT from ALN. (**B**) HPLC chromatogram of the crude extract obtained from the wild-type *A. alternata* FB1. Arrows indicate the peak positions of AOH and AME. (**C**) HPLC chromatogram of the crude extract obtained from the *omtI* knockout mutant of *A. alternata* FB1. The arrow indicates the peak position of AOH.

Understanding the biosynthesis of a compound is essential to develop it as an effective drug. We next investigated the biosynthesis of AOH and AME through genetic means. Previous study has reported that the gene cluster for the biosynthesis of AOH and its derivatives spans ~15 kb and consists of genes encoding polyketide synthase I (PksI), O-methyltransferase (OmtI), an FAD dependent monooxygenase (MoxI), a short-chain dehydrogenase (SdrI), putative extradiol dioxygenase (DoxI), and a transcription factor (AohR) (21). Among them, *pksI* (encoding a polyketide synthase) controls the synthesis of AOH, and *omtI* (encoding an O-methyl transferase) controls the synthesis of AME. After searching the genome of *A. alternata* FB1, we found a potential gene cluster including all the above six genes for the biosynthesis of AOH and its derivatives in the genome of strain FB1 (Fig. 9A). When comparing the two biosynthetic gene clusters, we found that the total length of gene cluster present in strain FB1 is ~17 kb, which is about 2 kb longer than the previously reported one. To verify the biosynthesis of AOH and AME in strain FB1, we successfully constructed the genetic operation system of *A. alternata* FB1 after much effort. Given that AME has a narrower inhibition spectrum and lower antibacterial capability than those of AOH, we next sought to delete the gene *omtI* from the genome of *A. alternata* FB1 to block the generation of AME. After the deletion of *omtI*, the crude extract still kept the inhibition activity against MRSA. After separation by HPLC, only a sharp peak was generated at the location of AOH of 11.944 min (Fig. 9C), which was verified by the spectrum as AOH; in contrast, the corresponding peak of AME almost totally disappeared (Fig. 9B and C). The above results verified that OmtI directed the biosynthesis of AME and the deletion of *omtI* gene could effectively block the generation of AME, which decreases the contamination of AME during the purification of AOH and facilitates us to efficiently obtain pure AOH in the future.

## DISCUSSION

In this study, the marine fungus *A. alternata* FB1 was found to produce the main secondary metabolites AOH and AME (Fig. 1), which exhibit significant inhibitory effects against MRSA (Fig. 1 and 2), a multidrug-resistant pathogen infecting human beings. AOH also possesses a broad spectrum of antibacterial activity, notably showing strong inhibition against the representative Gram-negative bacterium *V. anguillarum* (Fig. 3), a notorious aquaculture pathogen. Importantly, AOH and AME also effectively prevent zebrafish from MRSA and *V. anguillarum* infection *in vivo* (Fig. 7). Meanwhile, AOH and AME do not show any toxic effects on the normal human liver cell line under the concentrations exhibiting significant antibacterial activity against different pathogens (Fig. 8), strongly suggesting their great potentials in developing novel antibiotics for both human beings and aquaculture animals. Consistent with our proposal, scientific reports indicate that AOH, AME, and their other derivatives exhibit anticancer activity through several pathways, which facilitates them to be hopeful chemotherapeutic agents, though they were used to be regarded as toxic mycotoxin metabolites (22).

AOH and AME are typical polyketide natural products (21). Polyketide compounds are the largest class in microbial secondary metabolites and can inhibit bacteria through various mechanisms (46). Actually, many marine fungi can produce polyketide compounds with inhibitory activity against MRSA, *E. faecalis*, *B. cereus*, and even against viruses such as influenza and HIV (47). However, previous reports only simply tested the antibacterial activity of AOH and AME, with limited exploration of their specific antibacterial mechanisms. Here, in combination with electron microscopic observation, transcriptomic analysis, physiological and biochemical assays, as well as *in vivo* means, we detailedly disclosed the antibacterial mechanisms of AOH and AME. Based on our results, AOH and AME initially damage the cell membranes of MRSA or *V. anguillarum* (Fig. 3), leading to cell rupture, and thereby disturbing the cell’s internal energy metabolism (48). Consistently, the results of PI membrane permeability experiment confirmed that damage to the cytoplasmic membrane contributed to the antibacterial activity of AOH and AME (Fig. 5A). Indeed, some reports have indicated that certain antibiotics can alter the permeability of bacterial outer membranes to facilitate antibiotics’ entry into target sites (49).

Both AOH and AME could enhance ROS production and decrease the generation of hemolysin in pathogens (Fig. 5). ROS can cause oxidative damage to cell membranes, nucleic acids, and proteins, leading to structural and functional impairments (50). Actually, AOH and AME also effectively induced oxidative stress in human cancer cells and led to mitochondrial dysfunction-dependent cytotoxic effect (22), indicating increase in ROS production is a broad action means of AOH and AME toward both prokaryotic and eukaryotic pathogens. Hemolysins are toxins secreted by bacteria, which play a role in pathogenicity (34). For MRSA, there are α, γ, and δ hemolysins, along with a non-pore-forming β-hemolysin (35). It has been reported that α-hemolysin targeting drugs can reduce MRSA virulence by inhibiting its production, expression, and activity (34). In the case of *V. anguillarum*, there are six hemolysin genes: *vah1, vah2, vah3, vah4, vah5*, and M3 strain hemolysin gene. In the future, significant effort should be made to determine which type of hemolysin is the essential target of AOH and AME for the development of high-potency drugs.

Notably, both AOH and AME could effectively disorder pathogens’ cell division process. Indeed, there are many antibiotics that inhibit bacterial proliferation by interfering cell division step (33, 51–53). As is well known, cell division is a critical process for cell proliferation (54, 55). Under electron microscopic observation, the cell division processes of MRSA and *V. anguillarum* were obviously disordered by the treatment of AOH or AME (Fig. 3). Consistently, the transcriptomic results showed that the expression levels of key proteins involved in cell division (Fig. 4A through C), including FtsL, FtsQ, FtsZ, FtsA, and GpsB, were significantly changed, which were further confirmed by the qRT-PCR results (Fig. 4D). FtsZ plays a crucial role in bacterial cell cytoplasmic division, as it can aggregate to form a ring-shaped structure called the Z-ring, which binds to the inner membrane, and GpsB stabilizes the Z-ring (56). The protein complex FtsB-FtsL-FtsQ (FtsBLQ) is an essential component during cell division, primarily guiding and assembling peptidoglycan synthesis required for cell wall formation during division (57). The FtsA protein anchors FtsZ aggregates to the cytoplasmic membrane (58). Interestingly, AOH was found to arrest the cell cycles of several cancer cell lines in the G0/G1 phase or G2/M phase (22), suggesting cell division process might be a key target of AOH and its derivatives. Consistently, we demonstrated that AOH might target MRSA’s topoisomerases (Fig. 6), which are closely related to bacterial DNA replication and cell division (46). Specifically, bacterial topoisomerases IIA differ significantly in structure from their mammalian counterparts, allowing antimicrobial agents to selectively affect bacterial cells without harming mammalian cells (59). This makes topoisomerase IIA become a promising target for a range of antimicrobial agents.

Moreover, based on previous report of terrestrial *A. alternata* strain ATCC66981, we verified the existence and function of the gene cluster in charge of the biosynthesis of AOH and AME through bioinformatics and genetics means (Fig. 9). The gene cluster consisted of a polyketide synthase gene *pksI*, an O-methyltransferase gene *omtI*, an FAD-dependent monooxygenase gene *moxI*, a short chain dehydrogenase gene *sdrI*, a putative extradiol dioxygenase gene *doxI,* and a transcription factor gene *aohR*. When the gene cluster shown in *A. alternata* strain ATCC66981 (21) is compared, the protein sequence and cluster composition responsible for AOH and AME biosynthesis in marine fungus *A. alternata* FB1 are highly homologous, indicating the biosynthesis pathway is very conserved in both terrestrial and oceanic *A. alternata* strains. Notably, the only structural difference between AOH and AME is the presence of a diethyl ether group at the ninth carbon position in AME, added by the O-methyl transferase OmtI. Despite this minor structural change, the overall antibacterial activity and spectrum of AME are much weaker than those of AOH. Moreover, the deletion of *omtI* could effectively block the generation of AME, which is helpful for us to efficiently obtain purer AOH. These findings inspire us to perform more careful genetic studies for a better understanding of the biosynthesis and regulatory mechanisms of AOH and its derivatives in the future, which will facilitate us to find more promising drug candidates with higher activity and less toxicity.

In addition to potential antibiotics (e.g., AOH and AME), the marine fungus *A. alternata* FB1 is also capable of producing enzymes that efficiently degrade polyethylene (30). For the long-term goal, we try to construct a large-scale fermentation container for plastic degradation. This process will generate a huge amount of fungal biomass, which will result in tremendous energy waste if we cannot efficiently utilize these fermentation substances. Given that *A. alternata* FB1 is able to produce potential antibiotics, it will be a good idea to ingeniously connect plastic degradation with antibiotic extraction for both environmental pollution control and drug production in the future.

## MATERIALS AND METHODS

### Extraction of metabolites from *Alternaria alternata* FB1

The marine fungus *Alternaria alternata* FB1 was inoculated in the rice medium (containing rice 250 g, corn steep liquor 1 g, peptone 1.5 g, yeast extract 2.5 g, monosodium glutamate 3 g, and filtered seawater 500 mL). Before extracting the bioactive substances, strain FB1 was cultured in the rice medium at 28°C for 30 days. Thereafter, the cultures together with the rice medium were soaked in 500–750 mL of ethyl acetate for 1 week. Then, the ice residues were filtered out and the soaked liquid was retained, and the soaked liquid was evaporated at 50°C until dry. Finally, the remaining solid was re-dissolved in methanol to obtain a crude extract of fungal metabolites.

### Antibacterial assays

To screen for metabolites from *A. alternata* FB1 with antibacterial activity against pathogens, four Gram-positive pathogens (including methicillin-resistant *S. aureus*, *L. monocytogenes*, *E. faecalis*, and *B. cereus*) and four Gram-negative pathogens (including *P. aeruginosa* PAO1, *V. splendidus*, *V. anguillarum*, and *V. alginolyticus*) were selected as indicator strains. The antibacterial activity of metabolites from *A. alternata* FB1 was determined according to the previous report (60) with some modifications. In brief, solid culture media suitable for the growth of each bacterium (e.g., LB medium for MRSA, PAO1 strain, and *B. cereus*; TSB medium for *Listeria*; and BHI medium for *E. faecalis*) were melted, cooled to about 37°C, and inoculated with 0.5% overnight seed culture of the above pathogens. The LB medium contains 10 g/L peptone, 5 g/L yeast extract, and 10 g/L NaCl in 1 L of filtered distilled water, pH adjusted to 7.0; the BHI medium contains 10 g/L tryptone, 17.5 g/L beef heart extract powder, 5 g/L NaCl, 2 g/L glucose, and 2.5 g/L disodium hydrogen phosphate dodecahydrate in 1 L of filtered distilled water, pH adjusted to 7.4; the TSB medium contains 17 g/L tryptone, 3 g/L peptone, 5 g/L NaCl, 2.5 g/L dipotassium hydrogen phosphate, and 2.5 g/L glucose in 1 L of filtered distilled water, pH adjusted to 7.3. For *Vibrio* strains, about 100 µL of overnight culture was uniformly coated on a 2216E agar plate (containing 5 g/L tryptone and 1 g/L yeast extract in 1 L filtered seawater, pH adjusted to 7.4–7.6). If necessary, 10 g agar was added to the above liquid media to make the solid ones. Then, symmetrical holes were drilled 3 cm away from the center of the agar plate, and equal amounts of crude extract and methanol were added into different holes, respectively. The plate was incubated at 28°C for 48 h. The inhibition effects of bacterial growth were identified by visual observation of the presence or absence of a bacterial layer.

### Isolation and purification of antibacterial substances from *A. alternata* FB1

To obtain specific antibacterial substances from the crude extract produced by *A. alternata* FB1, MRSA was mainly used as the indicator bacterium to trace the antibacterial components. The methanol-dissolved extract was filtered through a 0.22 µm nylon membrane, and the crude extract was separated by molecular sieve, with the molecular sieve filler consisting of the Sephadex LH-20 (Solarbio, China). The mobile phase was anhydrous methanol, and 1.5 mL was collected every minute. The components separated by molecular sieve were tested for bacteriostasis, and the active parts were collected and concentrated. The concentrated active substances were purified by RP-HPLC (Agilent 1260, USA), using Eclipse XDB-C18 column (5 µm; 4.6 × 250 mm; Agilent, USA). The column was eluted at a flow rate of 2 mL/min with mobile phase A and mobile phase B under the following conditions: 0–5 min, 0% mobile phase B to 80% mobile phase B, and 5–20 min, 80% mobile phase B to 100% mobile phase B, wherein mobile phase A was composed of water and methanol (90:10, vol/vol) and mobile phase B was 100% methanol. The elution was monitored using a UV detector set at 260 nm. The inhibitory activity against the indicator bacteria was detected.

### Primary mass spectrometry analysis of antibacterial substances

The mass spectra of active antifungal substances were analyzed by quadrupole time-of-flight mass spectrometry (maXis plus, Bruker, Germany). The product obtained from liquid-phase purification was directly used in injection mode. The condition data of HCDMS/MS are ESI, spray voltage is 3 kV, ion transfer capillary temperature is 200°C, the dry gas is nitrogen, and the HCD collision gas was helium, in cationic mode. The collision energy of HCD is 30–40 eV. The experimental data were analyzed by DataAnalysis (Bruker, Germany).

### Nuclear magnetic resonance spectral analysis of bioactive compounds

The purified compound (~5 mg) was dissolved in deuterium reagent DMSO-d6, and the nuclear magnetic spectra of ^1^H-NMR (500 MHz, DMSO-d6) and ^13^C-NMR (125 MHz, DMSO-d6) were determined by Agilent Pro Pulse500MHz nuclear magnetic resonance spectrometer (Agilent, USA).

### Determination of the antibacterial activity of purified compounds

To determine the antibacterial activity of the purified antibacterial agents, growth inhibition tests were performed with the same eight pathogenic bacteria used for the activity analyses of crude extract of strain FB1 following the protocols described previously (61). Briefly, the biomass growth was measured by 24-well plates, and the total volume of bacterial solution and bacteriostatic substance added to each well was 1 mL. The above-mentioned pathogens were cultured overnight (at 28°C, 200 rpm) as the seed culture, which was inoculated at 0.5% concentration into the respective growth medium suitable for each pathogen. AOH at final concentrations of 1, 2, 5, or 8 µg/mL was used to treat MRSA; AOH at final concentrations of 2, 5, 8, or 10 µg/mL was used to treat *B. cereus*; AOH at final concentrations of 2, 5, 8 or 10 µg/mL was used to treat *E. faecalis*; AOH at final concentrations of 2, 5, 8, or 10 µg/mL was used to treat *L. monocytogenes*; AOH at final concentrations of 2, 5, 8, or 10 µg/mL was used to treat *V. splendidus*; AOH at final concentrations of 2, 3, 5, or 10 µg/mL was used to treat *V. anguillarum*. AME at final concentrations of 2, 5, 8, or 15 µg/mL was used to treat MRSA. The growth of MRSA, *B. cereus*, *L. monocytogenes,* and *E. faecalis* was observed after culturing at 37°C, 160 rpm for 24 h, while *V. splendidus* and *V. anguillarum* were cultured at 28°C, 160 rpm for 24 h. An equivalent amount of methanol was added to the control group. Each group was run in triplicate and repeated three times (60).

### Ultrastructure and morphological observation of pathogens treated by purified antibacterial substances

To investigate the effects of AOH on MRSA and *V. anguillarum* as well as AME on MRSA, SEM and TEM were employed to observe the morphological changes induced by different concentrations of antibacterial agents (62). The bacterial strains of MRSA and *V. anguillarum* were, respectively, cultured overnight at 37°C and 28°C with constant shaking at 160 rpm to obtain seed cultures. These seed cultures were then inoculated into the culture media suitable for the growth of respective pathogenic bacteria, with an inoculation volume of 0.5%. The cultures were divided into three groups, each consisting of three parallel subgroups: low-concentration treatment group, high-concentration treatment group, and control group. All groups were incubated at 37°C or 28°C with constant shaking at 160 rpm for approximately 3–4 h. When the bacterial cultures reached an optical density (OD_600_) of about 0.3, they were treated with different concentrations of AOH or AME.

For the treatment of MRSA with AOH, the final concentrations were 10 and 20 µg/mL for the low-concentration and high-concentration treatment groups, respectively. For the treatment of *V. anguillarum* with AOH, the final concentrations were 5 and 10 µg/mL for the low-concentration and high-concentration treatment groups, respectively. For the treatment of MRSA with AME, the final concentrations were 6 and 17 µg/mL for the low-concentration and high-concentration treatment groups, respectively. The control group was treated with an equivalent amount of methanol. Each experiment was conducted with three parallel groups, and the experiment was repeated three times. Once the OD_600_ value of bacterial cultures in the control group reached about 1.0, the cultures from all the above nine groups were centrifuged at 3,000 *g* for 10 min to remove the supernatant. The bacterial cells were washed twice with phosphate-buffered saline (PBS) and then fixed with 2.5% glutaraldehyde.

For SEM observation, the fixed cells were washed with a 10 mM phosphate buffer three times for 10 min each and subsequently dehydrated using an ethanol gradient. The samples were then coated with a thin layer of gold and platinum using a Hitachi MC1000 ion sputter coater (Hitachi, Japan) for 5 min. SEM observations were performed using a Hitachi S-3400N scanning electron microscope (Hitachi, Japan), with an operating voltage of 5 kV, a beam energy of 5 keV, and an exposure time of 30 seconds. In the case of TEM observation, samples that required slicing were embedded in plastic resin Epon 812, and ultrathin sections were prepared using a Leica EM UC7 ultramicrotome (Leica, Germany). These sections were stained with uranyl acetate and lead citrate and then observed using a Hitachi HT7700 transmission electron microscope (Hitachi, Japan) at 120 kV.

### Transcriptome analysis of pathogens treated with AOH or AME

For transcriptome analysis, the cultures of MRSA and *V. anguillarum* were prepared and treated with AOH or AME with the same procedures performed for electron microscopic assays described above. Thereafter, the AOH- and AME-treated MRSA cultures and the AOH-treated *V. anguillarum* culture were centrifuged to collect the bacterial cells, washed by PBS, and then frozen directly with liquid nitrogen and transported in dry ice to Novegene Company (Tianjin, China) for transcriptome analysis. For differential expression analysis, the *P* values were adjusted using the Benjamini and Hochberg method. A corrected *P*-value of 0.005 and log_2_ (fold change) of 1 were set as the threshold for significant differential expression. The detailed procedure for transcriptome analysis is described in the supplemental material.

### Detection of changes in cell integrity, hemolysis, and ROS production in the pathogens treated with AOH or AME

For propidium iodide membrane permeability assay, AOH at final concentrations of 1, 4, 8, or 10 µg/mL was added to the flasks containing the corresponding medium supplemented with the same amount of seed culture of MRSA. Likewise, AME at final concentrations of 1, 4, 8, or 12 µg/mL was added to the flasks containing the corresponding medium supplemented with the same amount of seed culture of MRSA. Thereafter, these cultures were incubated at 37°C with agitation at 160 rpm for 10 h. On the other hand, AOH at final concentrations of 1, 2, 3, or 6 µg/mL was added to the flasks containing the corresponding medium supplemented with the same amount of seed culture of *V. anguillarum*. The cultures were inoculated at 28°C, 160 rpm, for 10 h. All experimental groups were set in three parallel settings and repeated three times. The same volume of methanol was added to the culture to perform as the control group. PI dye was added to the final culture at a concentration of 1 µg/mL and incubated for 5 min. Following incubation, the cells were washed twice with PBS and resuspended, and their absorbance values were measured using a microplate reader at an excitation wavelength of 535 nm and an emission wavelength of 615 nm (63).

Hemolysin detection was performed as described previously (34). First, red blood cells were prepared with fresh defibrillated sterile sheep blood. The sheep blood was centrifuged at 1,000 *g* for 10 min, and the supernatant was removed. The red blood cells were washed with physiological saline several times until the supernatant was clear. After the supernatant was removed, the excess red blood cells were taken and mixed with normal saline to make 0.5% red blood cells for further experiments. AOH at final concentrations of 2, 4, 5, or 10 µg/mL was added to the flasks containing the corresponding medium supplemented with the same amount of seed culture of MRSA. Similarly, AME at final concentrations of 3, 4, 5, or 10 µg/mL was added to the flasks containing the corresponding medium supplemented with the same amount of seed culture of MRSA. These cultures were incubated at 37°C with agitation at 160 rpm for 10 h. On the other hand, AOH at final concentrations of 2, 3, 4, or 5 µg/mL was added to the flasks containing the corresponding medium supplemented with the same amount of seed culture of *V. anguillarum* and then cultured at 28°C with agitation at 160 rpm for 10 h. Meanwhile, the same amount of methanol was added to the respective pathogenic cells, and the same incubation and operation were performed. All the experimental groups were set in three parallel settings and repeated three times. After incubation, the cultures were centrifuged at 6,000 *g* for 6 min, and the supernatant was collected. Then, 20 µL of red blood cells was added to a 96-well plate, and 100 µL of the above supernatant was added to each well. The positive control and negative control were added with 100 µL of 0.1% Triton X-100 and 100 µL of physiological saline, respectively. The plate was placed in a 37°C incubator and allowed to incubate for 2–4 h. After incubation, the plate was centrifuged at 3,500 *g*, and the supernatant was transferred to a clean 96-well plate. The optical density was measured at 540 nm using a microplate reader.

ROS detection was performed as described previously (50). Feed solutions of MRSA and *V. anguillarum* were cultured overnight and then diluted with physiological saline to an OD_600_ of 0.3. All operations were performed in the absence of light. To 1 mL of diluted solution, 1 µL of ROS probe was added. The mixture was wrapped in aluminum foil to prevent light and incubated at 37°C with agitation at 160 rpm for 1 h. The mixtures were centrifuged at 6,000 *g* for 6 min, washed twice with PBS, and then added to PBS to blow well. AOH at final concentrations of 1, 2, 5, or 20 µg/mL and AME at final concentrations of 1, 2, 5, or 20 µg/mL were added to the MRSA culture medium and cultured at 37°C, 160 rpm for 5 min. On the other hand, AOH at final concentrations of 1, 2, 3, or 5 µg/mL was added to the *V. anguillarum* culture medium and cultured at 28°C, 160 rpm for 5 min. After incubation, the light absorption value was detected by the enzyme label. The wavelength of excitation light is 488 nm, and the wavelength of absorption light is 525 nm.

### Real-time quantitative reverse transcription PCR analysis

MRSA and *V. anguillarum* cells were cultured and treated with AOH or AME with the same procedures performed for transcriptomic assays described above. Thereafter, bacterial cells were collected by centrifugation at 12,000 *g* for 10 min. The cell pellets were transferred to 1 mL microcentrifuge tubes. Total RNAs from each sample were extracted using the Trizol reagent (Solarbio, China), and the RNA concentration was measured using Qubit RNA Assay Kit in Qubit 2.0 Fluorometer (Life Technologies, CA, USA). Then, RNAs from the corresponding sample were reverse transcribed into cDNA, and the transcriptional levels of different genes were determined by qRT-PCR using SybrGreen Premix Low rox (MDbio, China) and the QuantStudioTM 6 Flex (Thermo Fisher Scientific, USA).

The transcription levels of genes related to cell division in MRSA (including *ftsL, ftsQ, ftzA, ftsZ,* and *gpsB*) and genes related to cell division and flagella formation (including *zapE, ftsL, ftsQ, flgP, flgB, flgC,* and *fliL*) and chemotaxis (including *cheA, cheV, cheW, cheD*) in *V. anguillarum* were measured using a fluorescent quantitative PCR reagent kit (under the same conditions as those used for transcriptional analysis). The PCR condition was set as follows: initial denaturation at 95°C for 3 min, followed by 40 cycles of denaturation at 95°C for 15 seconds, annealing at 60°C for 15 seconds, and extension at 72°C for 15 seconds. 16S rRNA gene was used as an internal reference, and the gene expression was calculated using the 2^-ΔΔCt^ method, with each transcript signal normalized to that of 16S rRNA. Transcript signals for each treatment were compared to those of the control group. Specific primers for the above genes are shown in Table S1.

### Identification of MRSA topoisomerase as the action target of AOH

To overexpress MRSA topoisomerases (including Top1, Top2, Top3, and Top4), their encoding genes *ILP78_00775*, *ILP78_00780*, *ILP78_08890*, and *ILP78_03260* were, respectively, cloned in the expression vector pET28a by using a ligation-independent cloning method (64). PCR primers used for cloning are shown in Table S2. The final expression vectors for the above topoisomerases were, respectively, transformed into competent cells of *E. coli* BL21(DE3) (TsingKe, China), and transformants were incubated in the LB broth supplemented with 50 µg/mL kanamycin at 37°C. Protein expression was induced at an OD_600_ around 0.6 with 0.1 mM of isopropyl-1-thio-β-D-galactopyranoside, and the cells were cultured for a further 20 h at 16°C. To purify the His-tagged topoisomerases, the supernatant obtained from the respective cell cultures was loaded onto a 5 mL prepacked HiTrapTM nickel column (GE Healthcare, USA), which was pre-equilibrated with 25 mL of binding buffer (150 mM NaCl, 50 mM Tris-HCl, and 10% glycerol, pH 8.0). The column was then successively washed with 25 mL of binding buffer and 25 mL of washing buffer (20 mM imidazole, 150 mM NaCl, 50 mM Tris-HCl, and 10% glycerol, pH 8.0), respectively. The fusion proteins were eluted on an AKTA purifier system (GE Healthcare, USA) by using a linear gradient with the elution buffer (500 mM imidazole, 150 mM NaCl, 50 mM Tris-HCl, and 10% glycerol, pH 8.0). The eluted proteins were checked by SDS-PAGE, and the fractions containing corresponding size to His-tagged topoisomerases were collected and dialyzed against storage buffer (20 mM NaCl, 150 mM Tris-HCl, and 10% glycerol, pH 8.0) for 4 h. The concentrations of His-tagged topoisomerases were quantified by a BCA kit (Solarbio, China). The unwinding activity of topoisomerase was conducted with the following components: 2 µL of plasmid pCT74 (0.6 µg/µL), 2 µL of 10× reaction buffer, and 10 µM Top 1, TOPc, TOP3, or TOP4 in 16 µL storage buffer. The composition of the 10× reaction buffer was 500 mM Tris-acetate, 1 M NaCl, and 25 mM MgCl_2_. The same amount of storage buffer instead of protein used for the above reaction was regarded as the negative control. The reaction was performed at 37°C for 30 min. To check the reversible effect of AOH on the unwinding activity of topoisomerase, we pre-incubated 10 µM Top 1, TOPc, TOP3, or TOP4 (in 15 µL) with 1 µg of AOH (in 1 µL) at 37°C for 5 min and then 2 µL of plasmid pCT74 (0.6 µg/µL) and 2 µL of 10× reaction buffer were added. The above reaction was performed at 37°C for another 30 min. Subsequently, we added the loading buffer and placed the samples on ice. Finally, 1.5% agarose gel electrophoresis was performed to check the linear or supercoiled forms of pCT74.

### Protection activity assays of AOH and AME against the infection of MRSA and *V. anguillarum* by using zebrafish as the *in vivo* model

To test the antibacterial activities of AOH and AME *in vivo*, zebrafish embryos (Shandong YiXiYue Biotechnology Co., Ltd, China) at 24-h post-fertilization were chosen. Healthy, transparent, and regular embryos were selected and distributed in 24-well plates with five embryos per well and incubated at 28°C in 1 mL of embryo water (65). Then, varying volumes (10–100 µL) of overnight cultured MRSA or *V. anguillarum* cells were added to the well to determine the appropriate amount at which MRSA or *V. anguillarum* could cause abnormal development of zebrafish embryos. Based on this method, we found that 60 or 70 µL of overnight cultured MRSA or *V. anguillarum* cells could evidently lead to abnormal development of zebrafish embryos in a 1 mL incubation system. To test the potential negative impact of AOH and AME on zebrafish embryos, a final concentration of 8 or 10 µg/mL of AOH and a final concentration of 10 or 15 µg/mL of AME were, respectively, incubated with zebrafish embryos at 28°C for 24 h, and then the development of zebrafish embryos was observed. To detect the antibacterial activity of AOH and AME *in vivo*, 60 µL of overnight-cultured MRSA cells was mixed with AOH (5 or 8 µg/mL) or AME (8 µg/mL or 15 µg/mL) and then added to a final volume of 1 mL of zebrafish embryo medium; similarly, 70 µL of overnight-cultured *V. anguillarum* cells was mixed with AOH (8 or 10 µg/mL) and then added to a final volume of 1 mL of zebrafish embryo medium. An equal amount of methanol was used to mix with the pathogenic cells and performed as the negative control. The above zebrafish embryos were incubated at 28°C for 24 h and changes in the zebrafish embryos were observed. All experiments were performed in triplicate with three parallels per group. Observations on embryo development were conducted using an inverted microscope (NIKON TS100, Japan) equipped with a digital camera, at a magnification of 40×.

### Toxic effects of AOH and AME on the human normal cell line

To evaluate whether AOH and AME, which effectively inhibit MRSA growth at their final concentration, have impact on the cell viability of the human normal liver cell line (Chang liver), Chang liver cells in the logarithmic growth phase were used. After cell counting, they were seeded at a density of 1.0 × 10^4^ cells per well in a 96-well cell culture plate and placed in a cell culture incubator at 5% CO_2_ and 37°C for 24 h. On the following day, AOH and AME at final concentrations of 2, 5, 10, and 15 µg/mL were, respectively, added to the wells of the 96-well plate, and an equivalent amount of methanol was added to the control wells. Each group had three replicates, and the plate was returned to the incubator for an additional 24, 48, or 72 h. After treatment, 20 µL of MTT solution (concentration of 5 mg/mL) was added to each well and incubated for 4 h in the incubator. Subsequently, 100 µL of a triplex solution (containing 10 g SDS, 5 mL isobutyl alcohol, and 0.1 mL 10 M HCl, dissolved in double-distilled water to a final volume of 100 mL) was added and allowed to incubate for an additional 6 h in the incubator. The absorbance at 570 nm was measured using a microplate reader. This experiment was repeated three times (45).

### Gene deletion in *A. alternata* FB1

To delete the *omtI* gene from the genome of *A. alternata* FB1, a homologous recombination method was employed (66). Due to the fact that the wild-type *A. alternata* FB1 is sensitive to 100 µg/mL hygromycin, we could therefore operate gene knockout in the medium containing hygromycin. About 500-bp sequence upstream of the *omtI* gene, the hygromycin resistance gene with a promoter sequence, and about 500-bp sequence downstream of the *omtI* gene were constructed in turn via overlapping PCR with the primer shown in Table S3 and used for the deletion of *omtI* gene from *A. alternata* FB1.

Next, the protoplast of *A. alternata* FB1 was prepared. Fungal mycelium of *A. alternata* FB1 was first inoculated in the mCDB medium at 28°C, 120 rpm for 2 days. The mCDB medium contains 40 g/L glucose, 1 g/L yeast extract powder, 0.5 g/L magnesium sulfate, 1 g/L monarkite, 0.25 g/L ammonium chloride, 0.25 g/L potassium chloride, and 1 g/L potassium dihydrogen phosphate in 1 L filtered seawater, pH adjusted to 7.4. Afterward, the culture was centrifuged at 4,000 *g* for 10 min, the supernatant was discarded, and 30 mL of osmotic protectant was added, followed by 1.5% cellulase, 1.5% snail enzyme, and 0.35% lywallzyme. The osmotic protectant contains 10 mM sodium hydrogen phosphate, 20 mM calcium phosphate, pH 6.0, and 1.1 mM calcium chloride, sterilized by filtration. The mixture was then incubated at 32°C, 120 rpm for 4 h. A 20 µL sample of the fungal liquid was then checked under an optical microscope. Once a significant number of protoplasts were visible, the fungal liquid was filtered through magic cloth. After filtering, the liquid was centrifuged at 2,500 *g* for 10 min, the supernatant was discarded, and the pellet was resuspended in 10 mL STC solution (containing 50 mM Tris, 50 mM calcium chloride, and 1.2 M sorbitol, pH 8.0). After another centrifugation at 2,500 *g* for 10 min, the supernatant was discarded, and the pellet was resuspended in 10 mL of STC solution. Protoplast density was ensured to reach 10^7^/mL, then 10% DMSO was added and stored at −80°C.

Fungal transformation was performed as previously described (67). First, 5–10 µg of the recombinant fragment was added to the protoplast, incubated in an ice bath for 20 min, and then 1 mL of PTC solution (containing 40% PEG4000, 1.2 M STC, sterilized by filtration) was added. After a 20-min incubation at room temperature, 10 mL of TB3 medium (containing 200 g/L sucrose, 3 g/L Tryptone, and 3 g/L yeast extract powder in 1 L of filtered distilled water, pH adjusted to 7.0) and 10 µL (100 µg/mL) of ampicillin were added. The mixture was then incubated overnight at 25°C, 80 rpm. During this period, the recombinant fragment would undergo homologous recombination with the genome for knocking out *omtI* gene. After overnight incubation, the mixture was centrifuged at 4,000 *g* for 10 min, the supernatant was discarded, and the remaining fungal mycelium was added to the molten TB3 agar medium (1% agar) supplemented with 100 µL (100 µg/mL) of hygromycin B and cooled to 50°C. After a 10-h incubation at 28°C, another layer of TB3 agar medium (1.5% agar) containing 100 µL (100 µg/mL) of hygromycin B was poured on top. The plate was incubated at 28°C for 2–3 days. After fungal transformants emerged, they were passaged five times on plates containing 100 µL (100 µg/mL) of hygromycin B. The genome was then extracted using a fungal genomic extraction kit and sequenced with the same primers (Table S3) to check if the *omtI* gene was knocked out. Knockout strains were cultivated, and the crude extract was obtained and dissolved in methanol as described above. After diluting the crude extract five times, it was tested with HPLC under the same conditions to check the production of AOH and AME.

### Statistical analysis

All data are expressed as means ± SD. Statistical significances are analyzed by two-tailed Student’s *t* test using SPSS 17.0 (IBM). Differences of *P* ≤ 0.05 were considered statistically significant (*P ≤ 0.05 and **P ≤ 0.01).

## Data Availability

The complete genome sequence of *A*. *alternata* FB1 presented in this study has been deposited in the GenBank database with the accession number PRJNA672824. The raw sequencing reads from the transcriptomics analysis have been deposited to the NCBI Short Read Archive (accession numbers: PRJNA1021003 and PRJNA1021033).

## SUPPLEMENTAL MATERIAL

Supplemental information aem.00058-24-s0001.pdf Supplemental methods, figures, and tables.

ASM does not own the copyrights to Supplemental Material that may be linked to, or accessed through, an article. The authors have granted ASM a non-exclusive, world-wide license to publish the Supplemental Material files. Please contact the corresponding author directly for reuse.
