# Supplementary material for: Antibacterial insights into alternariol and its derivative alternariol monomethyl ether produced by a marine fungus
Source: Appl Environ Microbiol. 2024 Mar 12;90(4):e00058-24. doi: 10.1128/aem.00058-24 (PMC11022538; doi:10.1128/aem.00058-24)
Supplement: Supplemental information — Supplemental methods, figures, and tables. [file aem.00058-24-s0001.pdf]

SUPPLEMENTAL INFORMATION

Antibacterial insights into alternariol and its derivative alternariol monomethyl ether produced by a marine fungus

Rongmei Li[1,2,3,5], Zhenjie Su[2,3,4,5], Chaomin Sun[2,3,4,5*], Shimei Wu[1*]

[1]College of Life Sciences, Qingdao University, Qingdao, China

[2]CAS Key Laboratory of Experimental Marine Biology & Center of Deep Sea Research, Institute of Oceanology, Chinese Academy of Sciences, Qingdao, China

[3]Laboratory for Marine Biology and Biotechnology, Qingdao Marine Science and Technology Center, Qingdao, China

[4]College of Earth Science, University of Chinese Academy of Sciences, Beijing, China

[5]Center of Ocean Mega-Science, Chinese Academy of Sciences, Qingdao, China

[*] Corresponding author

Shimei Wu Email address: shimeiwu2016@126.com

Chaomin Sun Email address: sunchaomin@qdio.ac.cn

SUPPLEMENTARY METHODS

A detailed procedure for transcriptomic sequencing analysis.

(1) Library preparation for strand-specific transcriptome sequencing. A total amount of 3 μg RNA per sample was used as input material for the RNA sample preparations. Sequencing libraries were generated using NEBNext® Ultra™ Directional RNA Library Prep Kit for Illumina® (NEB, USA) following manufacturer's recommendations and index codes were added to attribute sequences to each sample. rRNA is removed using a specialized kit that leaves the mRNA. Fragmentation was carried out using divalent cations under elevated temperature in NEBNext First Strand Synthesis Reaction Buffer (5×). First strand cDNA was synthesized using random hexamer primer and M-MuLV Reverse Transcriptase ($RNaseH^-$). Second strand cDNA synthesis was subsequently performed using DNA Polymerase I and RNase H. In the reaction buffer, dNTPs with dTTP were replaced by dUTP. Remaining overhangs were converted into blunt ends via exonuclease/polymerase activities. After adenylation of 3' ends of DNA fragments, NEBNext Adaptor with hairpin loop structure was ligated to prepare for hybridization. In order to select cDNA fragments of preferentially 150~200 bp in length, the library fragments were purified with AMPure XP system (Beckman Coulter, Beverly, USA). Then 3 μL USER Enzyme (NEB，USA) was used with size-selected, adaptor-ligated cDNA at 37 ℃ for 15 minutes followed by five minutes at 95 ℃ before PCR. Then PCR was performed with Phusion High-Fidelity DNA polymerase, Universal PCR primers and Index (X) Primer. At last, products were purified (AMPure XP system) and library quality was assessed on the Agilent Bioanalyzer 2100 system.

(2) Clustering and sequencing. The clustering of the index-coded samples was performed on a cBot Cluster Generation System using TruSeq PE Cluster Kit v3-cBot-HS (Illumia) according to the manufacturer's instructions. After cluster generation, the library preparations were sequenced on an Illumina Hiseq platform and paired-end reads were generated.

(3) Data analysis. Raw data (raw reads) of fastq format were firstly processed

through in-house perl scripts. In this step, clean data (clean reads) were obtained by removing reads containing adapter, reads containing ploy-N and low quality reads from raw data. At the same time, Q20, Q30 and GC content the clean data were calculated. All the downstream analyses were based on the clean data with high quality. Reference genome and gene model annotation files were downloaded from genome website directly. Both building index of reference genome and aligning clean reads to reference genome were used Bowtie2-2.2.3 (1). HTSeq v0.6.1 was used to count the reads numbers mapped to each gene. And then FPKM of each gene was calculated based on the length of the gene and reads count mapped to this gene. FPKM, expected number of Fragments Per Kilobase of transcript sequence per Millions base pairs sequenced, considers the effect of sequencing depth and gene length for the reads count at the same time, and is currently the most commonly used method for estimating gene expression levels (2).

(4) Differential expression analysis. Differential expression analysis of two conditions/groups (two biological replicates per condition) was performed using the DESeq R package (1.18.0) (3). DESeq provide statistical routines for determining differential expression in digital gene expression data using a model based on the negative binomial distribution. The resulting *P*-values were adjusted using the Benjamini and Hochberg's approach for controlling the false discovery rate. Genes with an adjusted *P*-value < 0.05 found by DESeq were assigned as differentially expressed. (For DEGSeq without biological replicates) Prior to differential gene expression analysis, for each sequenced library, the read counts were adjusted by edgeR program package through one scaling normalized factor. Differential expression analysis of two conditions was performed using the DEGSeq R package (1.20.0) (4). The *P* values were adjusted using the Benjamini & Hochberg method. Corrected *P*-value of 0.005 and $\log_2$ (Fold change) of 1 were set as the threshold for significantly differential expression.

(5) GO and KEGG enrichment analysis of differentially expressed genes. Gene Ontology (GO) enrichment analysis of differentially expressed genes was implemented by the GOseq R package, in which gene length bias was corrected (5).

GO terms with corrected *P* value less than 0.05 were considered significantly enriched by differential expressed genes. KEGG is a database resource for understanding high-level functions and utilities of the biological system, such as the cell, the organism and the ecosystem, from molecular-level information, especially large-scale molecular datasets generated by genome sequencing and other high-throughput experimental technologies (http://www.genome.jp/kegg/) (6). We used KOBAS software to test the statistical enrichment of differential expression genes in KEGG pathways.

References

1. Langmead B, Salzberg SL. 2012. Fast gapped-read alignment with Bowtie 2. *Nat Methods* 9:357-U54.
2. Trapnell C, Pachter L, Salzberg SL. 2009. TopHat: discovering splice junctions with RNA-Seq. *Bioinformatics* 25:1105-1111.
3. Anders S, Huber W. 2010. Differential expression analysis for sequence count data. *Genome Biol* 11.
4. Wang LK, Feng ZX, Wang X, Wang XW, Zhang XG. 2010. DEGseq: an R package for identifying differentially expressed genes from RNA-seq data. *Bioinformatics* 26:136-138.
5. Young MD, Wakefield MJ, Smyth GK, Oshlack A. 2010. Gene ontology analysis for RNA-seq: accounting for selection bias. *Genome Biol* 11.
6. Kanehisa M, Araki M, Goto S, Hattori M, Hirakawa M, Itoh M, Katayama T, Kawashima S, Okuda S, Tokimatsu T, Yamanishi Y. 2008. KEGG for linking genomes to life and the environment. *Nucleic Acids Res* 36:D480-D484.

SUPPLEMENTARY FIGURES

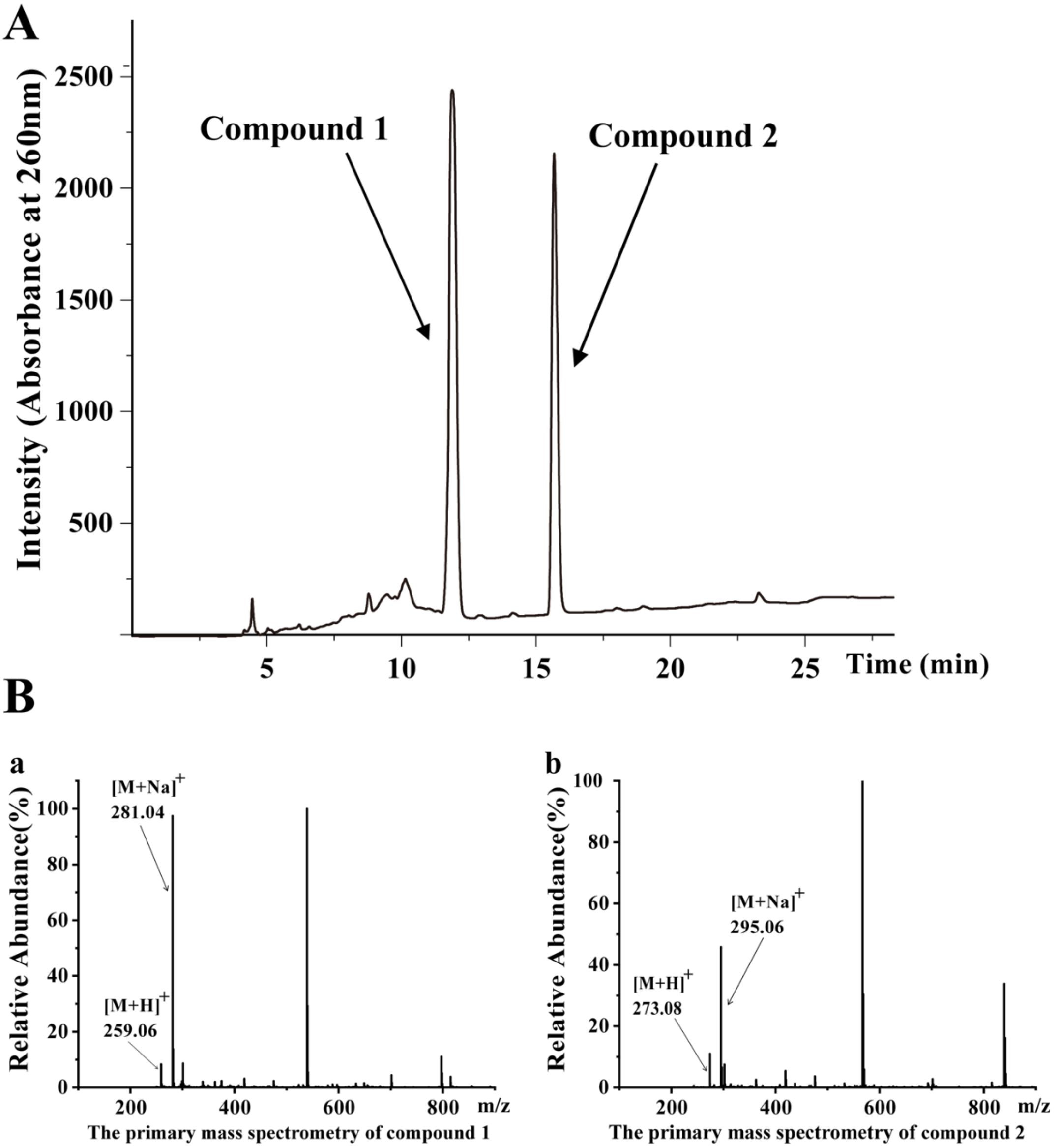

FIG S1 Structure elucidation of purified antibacterial agents. (A) The HPLC chromatogram of *A. alternata* FB1 fermentation crude extracts. The arrows indicate the peak positions of antibacterial agents. (B) Mass spectrometry analysis of the antibacterial agents eluted at 11.944 min (panel a) and 15.584 min (panel b) shown in Figure S1A.

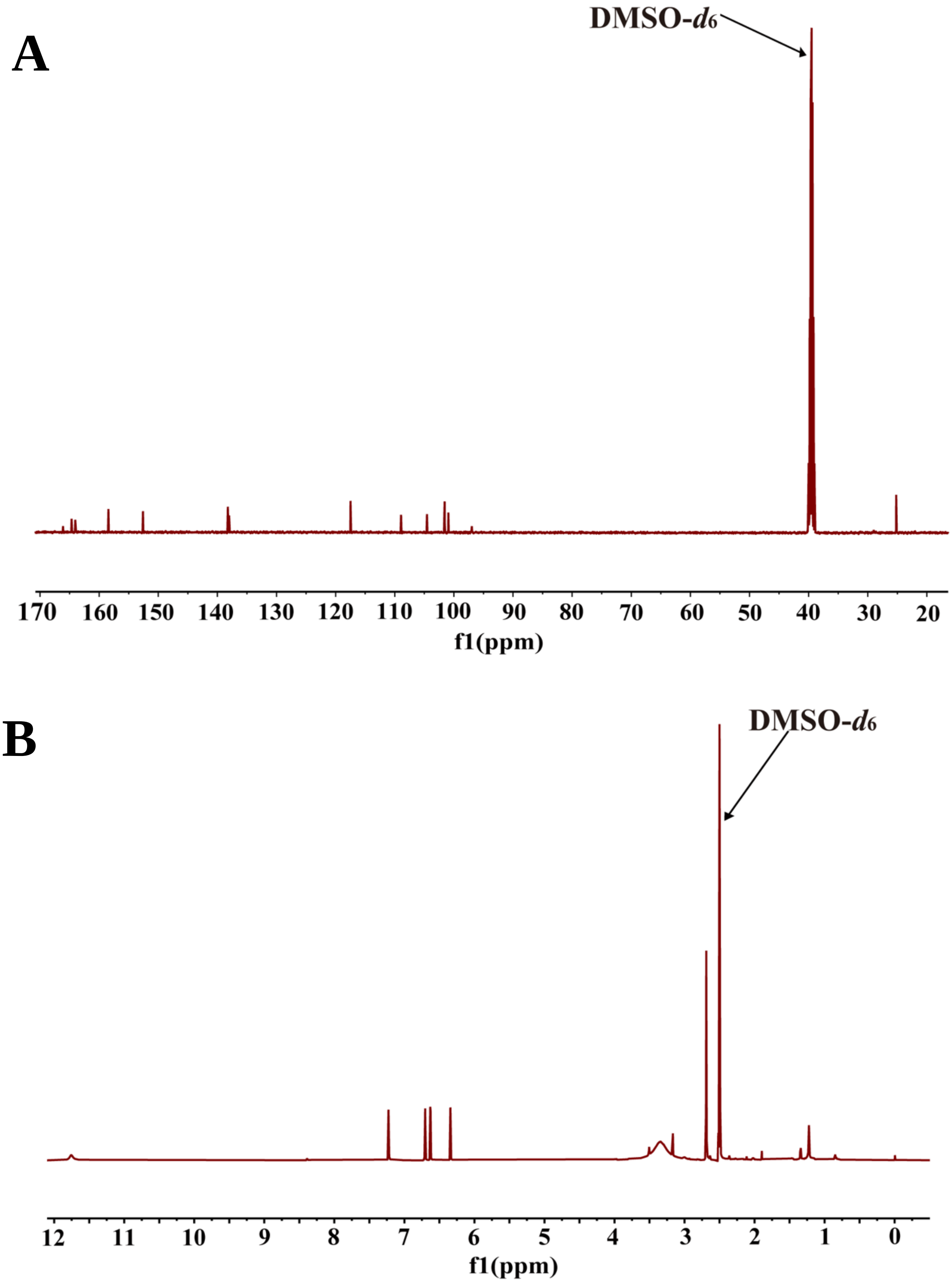

FIG S2 NMR spectra of AOH. (A) ^{13}C NMR spectrum of AOH. (B) ^{1}H NMR spectrum of AOH.

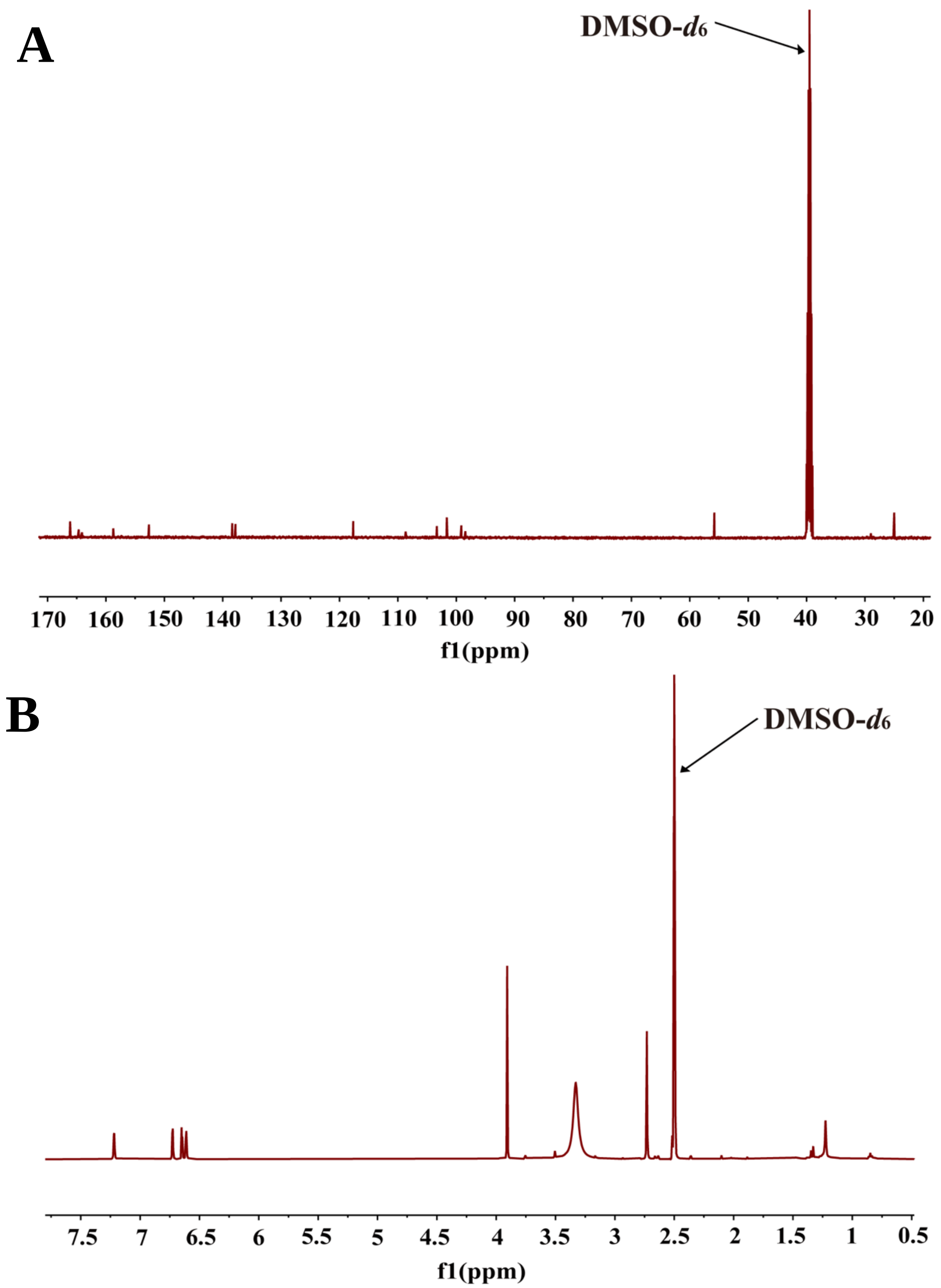

FIG S3 NMR spectra of AME. (A) ^{13}C NMR spectrum of AME. (B) ^{1}H NMR spectrum of AME.

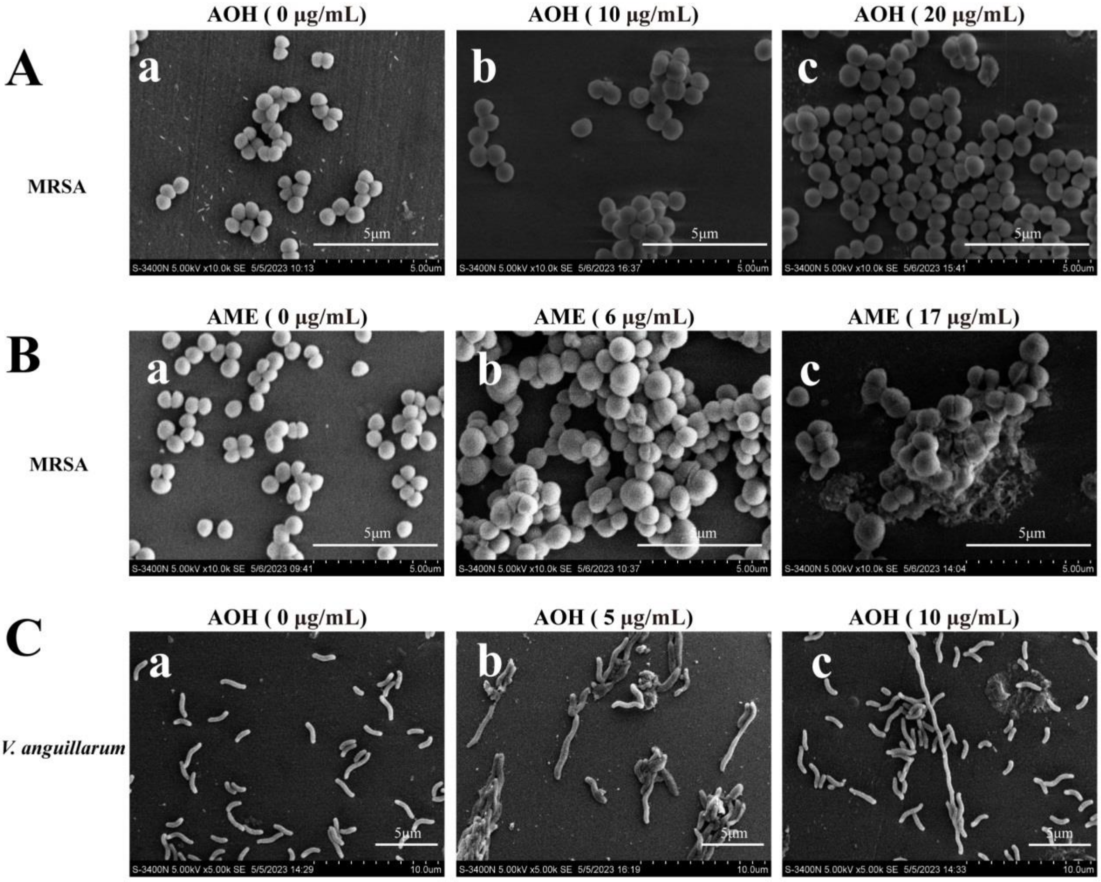

FIG S4 Scanning electron microscope (SEM) observation of different pathogens treated by AOH or AME. (A) SEM observation of MRSA treated by different concentrations of AOH. The same amount of methanol was used for control. (B) SEM observation of MRSA treated by different concentrations of AME. The same amount of methanol was used for control. (C) SEM observation of *Vibrio anguillarum* treated by different concentrations of AOH. The same amount of methanol was used for control. The final concentrations of AOH or AME used for assays were shown above each panel.

Table S1 Primers used in qRT-PCR

Primer name	Nucleotide Sequence (5'-3')
MRSA-16S-F	CGCGTGAGTGATGAAGGTCT
MRSA-16S-R	ATTCCGGATAACGCTTGCCA
240-F	CCGAAGCAACAACCACAAACT
240-R	TCTGCAATCTTTCCTCGCGTA
220-F	AGTCGACAACGAACAACCTCA
220-R	CGCTTACGTCTTAACTTCCGC
215-F	TGCGTAGAAGCATGTGGTGT
215-R	GCAACTTGCGTAACGTCTTCA
210-F	CACGTGGTTTAGGAGCAGGA
210-R	TACCAGTTCCGCCACCCATA
850-F	TGGCTCGTGGCTATAGAAGAGA
850-R	AGGTCTTGATGTTGCTACACG
570-F	TACCGTAACGGCTTACAGCG
570-R	CTCGTCACCAACCAATTGCC
490-F	CCATCATTCTAGGCAGGCGA
490-R	AAGATTCTGCACGCGGCTAT
445-F	GTGTACAGGTCATCCCGTGG
445-R	TTCCTTGCGGGCCATAAAGT
515-F	AACTGGGCGTAGTGTGGATG
515-R	TTCAGCACGACCACTCACTC
485-F	GCAAGCATTGGTCTTAGCCG
485-R	TTGCCATCACCCGTATCAGG
480-F	CGGTGTTTGGTGCAGAGTTAAG
480-R	GCAAGTGGGTGATCTGGGTTA
560-F	TAGAGCCGGATCTCACCACC
560-R	TCGACGATGGCATCACGAAT
710-F	TCGATTTGGCATGGAGGCTT
710-R	CCCTGGCCAATCACATCACT
495-F	TGCACTGGGAAGCCATTCTT
495-R	ACCGGAGAAATCTCTGCCAA
535-F	GTCAACCTAGCTCGGCGTTT
535-R	CACCTAGGTCTTGACGCTGTT
525-F	GGTTCGTGCGTTGCCATTAC
525-R	AAGTACGCCACAACATCGCT

Table S2 Primers used for topoisomerase expression

Primer name	Nucleotide Sequence (5'-3')
00775-MRSA-F	TGGGTCGCGGATCCGAATTCATGGCTGAATTACCTCAATCAAGAATAAATGAAC
00775-MRSA-28-R	GATTGAGGTAATTCAGCCATGAATTCGGATCCGCGACCC
00775-MRSA-28-F	AACAATCATCAGATGAAGAACTCGAGCACCACCACCACCA
00775-mrsa-R	TGGTGGTGGTGGTGCTCGAGTTCTTCATCTGATGATTGTTGTATATCTTCTTCAAC
00780-MRSA-F.	GCGGATCCGAATTCGAGCTCATGGTGACTGCATTGTCAGATGT
00780-MRSA-28-R	TCTGACAATGCAGTCACCATGAGCTCGAATTCGGATCCGC
00780-MRSA-28-F	TTTATGCAAACTTAGACTTCCTCGAGCACCACCACCACCA
00780-mrsa-R	TGGTGGTGGTGGTGCTCGAGGAAGTCTAAGTTTGCATAAACTGCATTATCTTCT
03260-mrsa-F	GCGGATCCGAATTCGAGCTCATGAAATCTTTAATATTAGCTGAAAAACCATCAGTCG
03260-MRSA-28-R	GCTAATATTAAAGATTTCATGAGCTCGAATTCGGATCCGC
03260-MRSA-28-F	CATTAAAGAACTTGAATTTACTCGAGCACCACCACCACCA
03260-mrsa-R	TGGTGGTGGTGGTGCTCGAGTAAATTCAAGTTCTTTAATGCATCTTTAAACGGATTATT
008890-mrsa-F	GCGGATCCGAATTCGAGCTCATGACATTGGCAGATAATTTAGTCATTGTTGA
008890-MRSA-28-R	AAATTATCTGCCAATGTCATGAGCTCGAATTCGGATCCGC
008890-MRSA-28-F	ATAAAGAGGCAGCGCAGAAACTCGAGCACCACCACCACCA
008890-mrsa-R	TGGTGGTGGTGGTGCTCGAGTTTCTGCGCTGCCTCTTTATAATCG

Table S3 Primers used for gene knockout

Primer name	Nucleotide Sequence (5'-3')
hyg-F	GTCGACGTTAACTGATATTGAAGGAGC
hyg-R	CTATTCCTTTGCCCTCGGACGA
007381-down-F	GTCCGAGGGCAAAGGAATAGTGTTGCATGACTGGTGTTTCGC
007381-down-R	AAGGATATATTGGCTGTCTTGAGTTGGTG
007381-up-F	CTCTACCTCTGCACCTTGATAGTGG
007381-up-R	CAATATCAGTTAACGTCGACGTTGCACGAAGAGCTGAAGGGTA
